# Isolation of a novel human prion strain from a *PRNP* codon 129 heterozygous vCJD patient

**DOI:** 10.1371/journal.ppat.1012904

**Published:** 2025-02-20

**Authors:** Fuquan Zhang, Susan Joiner, Jacqueline M. Linehan, Florin Pintilii, Tamsin Nazari, Fabio Argentina, Connor Preston, Maged Taema, Thomas J. Cunningham, Emmanuel A. Asante, Tzehow Mok, Simon Mead, Sebastian Brandner, John Collinge, Jonathan D.F. Wadsworth

**Affiliations:** 1 MRC Prion Unit at UCL, Institute of Prion Diseases, University College London, London, United Kingdom; 2 National Prion Clinic, National Hospital For Neurology and Neurosurgery, University College London NHS Foundation Trust, London, United Kingdom; 3 Department of Neurodegenerative Disease, UCL Queen Square Institute of Neurology and Division of Neuropathology, the National Hospital For Neurology and Neurosurgery, University College London NHS Foundation Trust, London, United Kingdom; Creighton University, UNITED STATES OF AMERICA

## Abstract

The epizootic prion disease of cattle, bovine spongiform encephalopathy (BSE), caused variant Creutzfeldt-Jakob disease (vCJD) in humans following dietary exposure. Codon 129 polymorphism of the human prion protein gene (*PRNP*), encoding either methionine (M) or valine (V), dictates the propagation of distinct human prion strains and up to now all but one neuropathologically confirmed vCJD patients have had a 129MM genotype. Concordant with this genetic association, transgenic modelling has established that human PrP 129V is incompatible with the vCJD prion strain and that depending on codon 129 genotype, primary human infection with BSE prions may, in addition to vCJD, result in sporadic CJD-like or novel phenotypes. In 2016 we saw the first neuropathologically confirmed case of vCJD in a patient with a codon 129MV genotype. This patient’s neuropathology and molecular strain type were pathognomonic of vCJD but their clinical presentation and neuroradiological features were more typical of sporadic CJD, suggestive of possible co-propagation of another prion strain. Here we report the transmission properties of prions from the brain and lymphoreticular tissues of the 129MV vCJD patient. Primary transmissions into transgenic mice expressing human PrP with different codon 129 genotypes mainly produced neuropathological and molecular phenotypes congruent to those observed in the same lines of mice challenged with prions from 129MM vCJD patient brain, indicative that the vCJD prion strain was the dominant propagating prion strain in the patient’s brain. Remarkably however, some transgenic mice challenged with 129MV vCJD patient brain propagated a novel prion strain type which at secondary passage was uniformly lethal in mice of all three *PRNP* codon 129 genotypes after similar short mean incubation periods. These findings establish that cattle BSE prions can trigger the co-propagation of distinct prion strains in humans.

## Author summary

Bovine spongiform encephalopathy (BSE) prions from cattle caused variant Creutzfeldt-Jakob disease (vCJD) in humans following dietary exposure. Research with genetically altered mice (transgenic mice) enables investigation of the prion strains that may be generated when people become infected with BSE prions. These studies have indicated that a person’s genetic makeup may critically determine whether BSE prion infection causes the vCJD prion strain to develop or instead triggers the generation of alternative prion strains resulting in different patterns of disease. In 2016 we saw a patient who had typical vCJD pathology but a genetic makeup (129MV) and clinical symptoms different to all previous vCJD patients (129MM). Here we used transgenic mice to characterize what prion strains were generated in the 129MV vCJD patient. Consistent with the patient’s diagnosis we found that the vCJD prion strain was the dominant prion strain in the patient’s brain tissue, remarkably however, transmissions to our transgenic mice also revealed the presence of a previously unrecognised human prion strain. These findings establish that infection with cattle BSE prions can trigger the generation of distinct prion strains in humans.

## Introduction

Bovine spongiform encephalopathy (BSE) prions from cattle infected humans causing variant Creutzfeldt-Jakob disease (vCJD), provoking an animal and public health crisis [1,2]. While the incidence of vCJD has been in decline in the UK since its peak in 2000, all definite diagnoses up to 2016 have been in patients with a particular prion protein (PrP) genotype (MM) at polymorphic codon 129, where either methionine (M) or valine (V) is encoded [3]. Experience of other acquired human prion diseases, kuru and iatrogenic CJD [4,5], and extensive transmission studies in transgenic mice expressing human PrP argued that further cases might occur in the other *PRNP* codon 129 genotypes (VV and MV) with longer incubation periods and with different disease phenotypes [1,2,6–14]. Prior to 2016, a patient with a suspected diagnosis of vCJD and MV genotype, died without tonsil biopsy or confirmatory autopsy [15], and transmission of vCJD infection via blood transfusion was detected post-mortem in the spleen of an individual with the MV genotype who died from a non-neurological disorder [16,17].

In 2016 we identified the first definite vCJD patient with a codon 129MV genotype [18]. This patient had pathognomonic vCJD neuropathology and molecular strain type but a clinical presentation and neuroradiological features more typical of sporadic CJD, raising the possibility of co-propagation of another prion strain. Although additional cases will be needed to draw firm conclusions about any consistent change in phenotype of human BSE prion infection in the UK, this 129MV vCJD patient provided the first opportunity to study the distribution and strain properties of prions propagated in brain and peripheral lymphoreticular tissues in this genotype and to determine whether this is distinct from that seen in 129MM vCJD patients.

Using sensitive biochemical analyses of patient tissues and transmission studies to humanized transgenic and wild-type mice we aimed to address the following key questions:

(1) Whether 129MV vCJD has a peripheral pathogenesis similar to 129MM vCJD and whether 129MV vCJD poses any significant new risks for secondary transmission of human prions through iatrogenic routes; (2) Whether there is co-propagation of vCJD prions with sporadic CJD-like or novel prions strains in 129MV vCJD patient brain or lymphoreticular tissues; (3) Whether anonymous screening of surgical appendix tissue [19–21] is likely to have informed upon the prevalence of subclinical vCJD in *PRNP* 129MV individuals.

## Results

### Summary of historical transmissions of 129MM vCJD prions to transgenic and wild-type mice

Transgenic mice that overexpress one or other of the two common polymorphic forms of human PrP, with either M or V at residue 129, on a congenic mouse PrP null background are highly appropriate models for investigating human prion strain diversity. These mice can faithfully replicate different human prion strains with maintenance of the key neuropathological features and the biochemical properties of protease-resistant, disease-related PrP assemblies (PrP^Sc^). Following limited digestion with proteinase K and western blotting, prion strain-specific PrP^Sc^ types can be distinguished by differences in N-terminally truncated PrP proteolytic fragment sizes and the compositional ratios of the three PrP glycoforms (di-, mono- and non-glycosylated PrP) [2,12]. The transgenic mice used in this study have known susceptibility to a wide range of sporadic and acquired human prion disease isolates as well as to classical BSE prions from cattle [12,22,23].

Over the last twenty five years we have transmitted vCJD prions from multiple 129MM vCJD patient brain samples to transgenic mice expressing human PrP and wild-type FVB/N mice [6–9,23–25]. All 129MM vCJD isolates examined have behaved consistently in each line of mice and have shown prion transmission properties that readily distinguish the vCJD prion strain from all other forms of human prion disease. In the present study we have used these multiple historical 129MM vCJD prion transmissions as a control reference series with which to compare new transmissions of 129MV vCJD patient tissues. In all of these prior studies (summarized below and in S1 Table) we have never observed the spontaneous generation of prion infection in aged control groups of the humanized transgenic mouse lines or in wild-type FVB/N mice.

Challenge of our transgenic mice expressing human PrP 129M with 129MM vCJD patient brain results in a high incidence of prion infection and faithful propagation of type 4 PrP^Sc^ (London classification, [26]) [7–9,23–25] which is pathognomonic of the vCJD prion strain [8,26,27]. Propagation of type 4 PrP^Sc^ in these mice is often accompanied by the key neuropathological hallmark of vCJD, the presence of abundant florid PrP plaques in the cortex and other brain regions, which are frequently seen on a strong background of synaptic PrP deposition [7–9,23,24].

Notably, although vCJD prions produce high attack rates of infection in human PrP 129M transgenic mice (typically 100%), most mice do not develop clinical signs of prion disease and instead remain subclinically infected to advanced old age [7–9,23,24]. This lack of clinical end point has been observed with numerous vCJD brain isolates that we have examined, including those with very high levels of type 4 PrP^Sc^, and indeed, even with vCJD isolates that produce a high incidence of clinical prion disease the mean incubation periods are close to the lifespan of the mice. Similar long mean incubation periods (around 500 days) for primary transmission and secondary passage of vCJD prions have also been seen by other researchers in Tg650 transgenic mice that express human PrP 129M in brain at 3-times higher levels than our 129MM Tg35c mice [28,29]. This situation of prominent subclinical infection and highly prolonged clinical incubation times precludes our ability to reliably estimate vCJD prion titre in human PrP 129M mice using conventional serial dilution and incubation period methods [30]. Consequently, we have found that demonstration of vCJD prion transmission to human PrP 129M mice is most reliably determined by demonstrating the propagation of type 4 PrP^Sc^ in the brain of inoculated recipients by immunoblotting rather than measuring the incidence and timing of clinical prion disease [7,8,24,25,31].

In contrast to the efficiency with which 129MM vCJD prions infect human PrP 129M mice, primary challenge of transgenic mice expressing human PrP 129V is characterized by a transmission barrier and often only a proportion of inoculated mice become infected [6,8,10,11,25]. As with human PrP 129M mice, subclinical infection at advanced old age is a common feature of these transmissions. Infected vCJD-challenged human PrP 129V mice propagate a novel prion strain associated with type 5 PrP^Sc^ [6,8,10,25] which shares the same predominance of the diglycosylated PrP glycoform seen in type 4 PrP^Sc^ but is distinguished by proteinase K digestion products of greater molecular mass, indicative of a distinct PrP^Sc^ conformation [6,8,10,25]. Propagation of type 5 PrP^Sc^ following primary transmission of vCJD prions is generally associated with low levels of pathological PrP deposition in brain when visualized by immunohistochemistry (IHC). Abnormal PrP deposition (when detected at primary transmission) is observed as focal or patchy diffuse labelling mainly in the midbrain and brainstem [6] and occasionally in some mice, as large non-florid PrP plaques in the corpus callosum [8,10,25]. There is a notable absence of florid PrP plaques even after secondary passage of type 5 PrP^Sc^ isolates in further human PrP 129V mice [8]. As with human PrP 129M mice, the most reliable way of assessing vCJD prion transmission rates in human PrP 129V mice is through detection of PrP^Sc^ in the brain of inoculated recipients following long clinically silent survival periods.

In human PrP 129MV mice expressing a 2:3 ratio of human PrP 129M:129V, prions from 129MM vCJD patient brain produce high attack rates of infection (typically 100%) and faithful propagation of type 4 PrP^Sc^, however mice do not develop clinical signs of prion disease and remain subclinically infected to advanced old age [9]. Notably, type 4 PrP^Sc^ propagation does not result in typical vCJD neuropathology in these mice and instead is associated with large non-florid PrP plaques in the corpus callosum accompanied by diffuse synaptic PrP deposition and occasional small non-florid PrP plaques in the brainstem and thalamus [9]. We interpreted these findings to indicate that PrP V129 may have a dominant negative effect on florid PrP plaque formation and evolution of characteristic vCJD neuropathology in these heterozygous mice [9].

In wild-type FVB/N mice, vCJD prions from 129MM patient brain have a high primary transmission rate, usually resulting in clinical prion disease in infected mice, although incubation periods are prolonged (typically in the range of 300–400 days) [6,8,10,11,25]. In these transmissions a distinctive diglycosylated PrP dominant PrP^Sc^ type is propagated in brain which is identical to that seen after transmission of cattle BSE prions to the same mice [6,8,10]. Abnormal PrP deposition in infected mice consists mainly of diffuse and granular deposits with occasional small PrP plaques, however florid PrP plaques are not observed [8,25]. The efficiency with which vCJD prions transmit infection to wild-type FVB/N mice is remarkable and readily distinguishes the vCJD prion strain from all other prion strains that are propagated in other forms of human prion disease [6,8,10,11].

Importantly, the phenotypes seen in our transgenic mice following challenge with 129MM vCJD prions have been subsequently replicated in other lines of humanized transgenic mice generated in other laboratories [28,32], thereby demonstrating the highly reproducible transmission properties of 129MM vCJD prions in transgenic mice expressing 129M or 129V human PrP.

## The present study

### Primary transmissions of prions from 129MV vCJD patient frontal cortex to transgenic and wild-type mice

Frontal cortex from the 129MV patient showed pathognomonic vCJD neuropathology and type 4 PrP^Sc^ molecular strain type [18]. No evidence for co-propagation of other PrP^Sc^ types was detected by examining PrP^Sc^ fragment size or glycoform ratio on western blots [18] (S1 Fig). Type 4 PrP^Sc^ was also uniformly observed in parietal cortex, thalamus and cerebellum [18].

**Fig 1:**
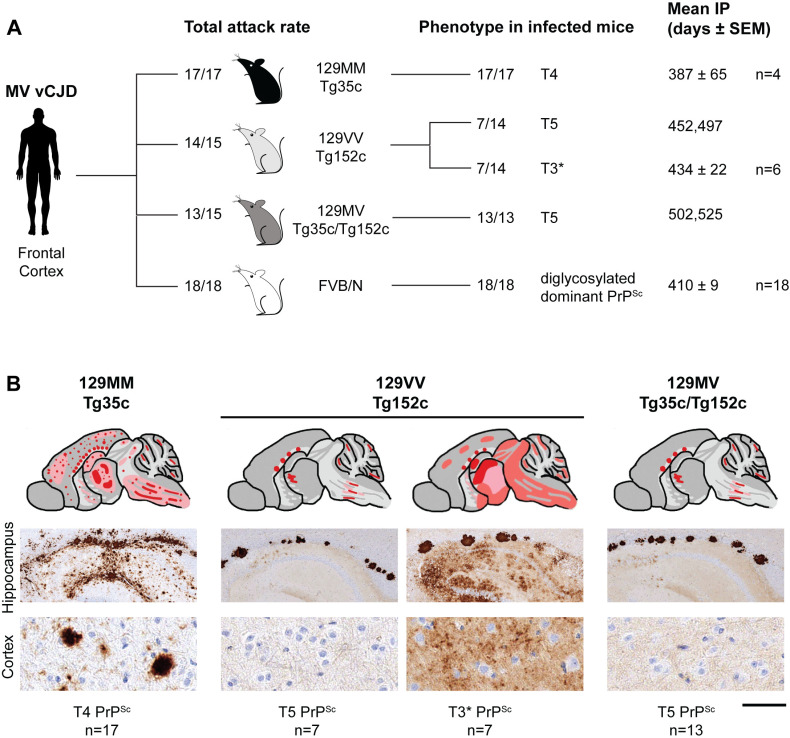
Prion transmission rates and findings in transgenic and wild-type mice challenged with 129MV vCJD patient frontal cortex. (A) Overview of transmission rates and molecular and neuropathological findings in recipient mice. Mice were intracerebrally inoculated with 1% (w/v) homogenate prepared from 129MV vCJD patient frontal cortex. Total attack rate is defined as the total number of clinically affected and subclinically infected mice as a proportion of the number of inoculated mice after post-inoculation periods extending to 600 days. Subclinical prion infection was assessed by immunohistochemical examination of brain for abnormal PrP deposition and western blot analyses of mouse brain homogenates for detectable PrP^Sc^ (by direct western blotting or after NaPTA precipitation if required). The type of PrP^Sc^ (PrP^Sc^ types 4, 5 and 3*; T4, T5, T3*) seen in infected mice is reported together with mean incubation periods (Mean IP) for clinically affected mice in days (where n ≥ 3 the mean ± SEM is reported otherwise individual incubation times are given). (B) PrP deposition patterns in infected transgenic mouse brains by IHC using anti-PrP monoclonal antibody 3F4. Upper panels show schematic drawings in which the overall spatial distribution and intensity of abnormal PrP deposition is indicated by red shading with PrP plaques shown as red dots. Lower panels show representative PrP IHC in hippocampus (including corpus callosum) and cortex. Scale bar, 300 µm for upper row (hippocampus) and 30 µm for lower row (cortex).

**Fig 2:**
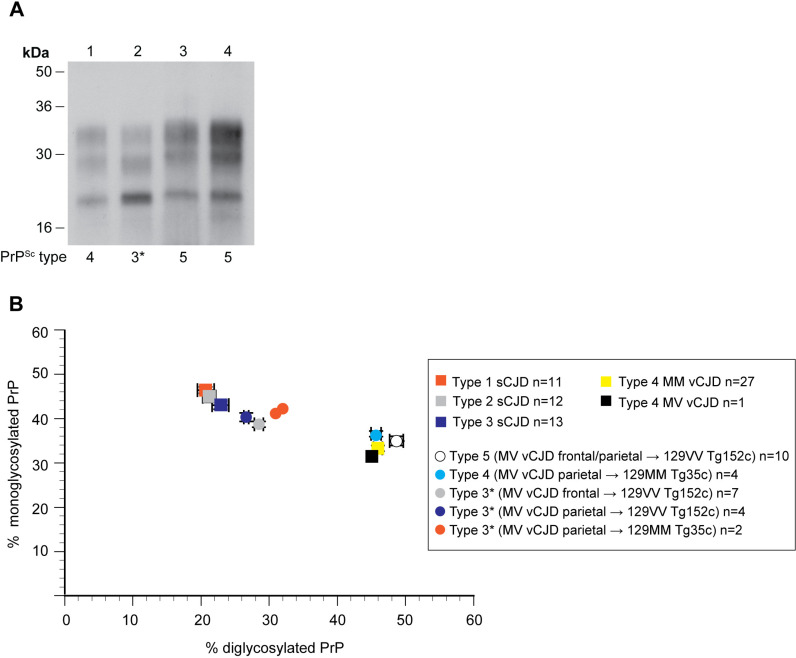
PrP^Sc^ types seen in the brain of transgenic mice after primary transmission of 129MV vCJD patient frontal or parietal cortex. (A) Western blot of proteinase K-digested 10% (w/v) brain homogenates from transgenic mice after challenge with frontal cortex from the 129MV vCJD patient using anti-PrP monoclonal antibody 3F4 and high sensitivity enhanced chemiluminescence. The propagated PrP^Sc^ types are shown below each lane. Lane 1, 129MM Tg35c brain, lane 2, 129VV Tg152c brain, lane 3, 129VV Tg152c brain, lane 4, 129MV Tg35c/Tg152c brain. (B) Ratios of the di- and mono-glycosylated protease-resistant PrP glycoforms in PrP^Sc^ from transgenic mouse brain after challenge with frontal or parietal cortex from the 129MV vCJD patient in comparison to PrP^Sc^ from the 129MV vCJD patient’s frontal cortex and reference cases of frontal cortex from patients with sporadic CJD (PrP^Sc^ types 1, 2 or 3; London classification, [26]) and 129MM vCJD patients with type 4 PrP^Sc^. Where sample size is ≥ 3 symbols show mean percentage ± SEM otherwise individual samples are shown. In some cases the error bars are smaller than the symbols used. Squares, human samples, circles, mouse primary transmission samples.

Type 4 PrP^Sc^ positive 1% (w/v) frontal cortex homogenate from the 129MV vCJD patient was intracerebrally inoculated into groups of 20 of each mouse line followed by post-inoculation observation periods extending to 600 days. Brains from mice were examined by histology (including PrP IHC) and biochemical analyses. Findings from these transmissions are summarized in S2 Table and Figs 1,2 and S2.

In 129MM Tg35c mice we saw a high incidence of infection (17/17 inoculated mice) but low incidence of clinical prion disease (4/17 infected mice) (S2 Table and Fig 1A). In all infected mice we observed the occurrence of typical vCJD neuropathology with abundant florid PrP plaques (Figs 1B and S2) and uniform propagation of type 4 PrP^Sc^ (Fig 2A). These findings were therefore entirely consistent with historical transmissions of 129MM vCJD patient brain to the same mice.

In 129MV Tg35c/152c mice, 13/15 inoculated recipients were infected (of which two developed clinical prion disease) and all infected mice propagated type 5 PrP^Sc^ accompanied by the typical neuropathology associated with this molecular strain type (prominent, irregular non-florid PrP plaques in the corpus callosum) (S2 Table and Figs 1 and 2). The uniform propagation of type 5 PrP^Sc^ in 129MV Tg35c/152c mice most likely reflects the 1:3 ratio of 129M:129V human PrP expressed in these mice. Previously, transmission of 129MM vCJD type 4 PrP^Sc^ to 129MV Tg45/152 mice expressing a 2:3 ratio of 129M:129V human PrP resulted in uniform propagation of type 4 PrP^Sc^ [9] (S1 Table).

**Fig 3:**
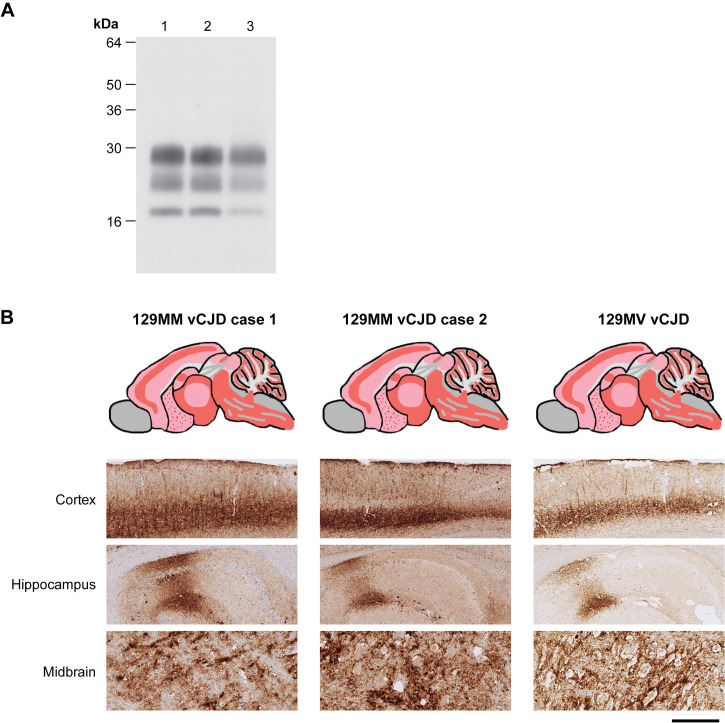
Molecular and neuropathological findings in wild-type FVB/N mice challenged with frontal cortex from vCJD patients with 129MM and 129MV genotypes. (A) Western blot of proteinase K-digested 10% (w/v) brain homogenates from FVB/N mice using anti-PrP monoclonal antibody 6D11 and high sensitivity enhanced chemiluminescence. Lanes 1 and 2, FVB/N mouse brains after challenge with 129MM vCJD patient frontal cortex from reference cases 1 and 2 respectively, lane 3, FVB/N mouse brain after challenge with 129MV vCJD patient frontal cortex. (B) PrP deposition patterns by IHC in the same transgenic mouse brains shown in panel A using anti-PrP monoclonal antibody 6D11. Upper panels show schematic drawings in which the overall spatial distribution and intensity of abnormal PrP deposition is indicated by red shading with PrP plaques shown as red dots. Lower panels show representative PrP immunohistochemistry in cortex, hippocampus and midbrain. Scale bar 300 µm for upper two rows (cortex and hippocampus) and 60 µm for lower row (midbrain).

In wild type FVB/N mice, 18/18 inoculated recipients were infected and all developed clinical prion disease (Fig 1A and S2 Table). All mice showed the propagation of the same diglycosylated PrP dominant PrP^Sc^ type (Fig 3A) and patterns of PrP deposition (Fig 3B) that mirror transmissions of prions from 129MM vCJD patient brain to the same mice (Fig 3A and 3B).

While transmissions of 129MV vCJD frontal cortex to 129MM and 129MV lines of transgenic mice and to wild-type FVB/N mice were entirely consistent with findings from transmissions of 129MM vCJD patient brains to the same mice, in transmissions to 129VV Tg152c mice we observed something quite different. Here a proportion of inoculated mice had a neuropathological and molecular phenotype that we have never previously seen following challenge with brain samples from multiple 129MM vCJD patients or patients with various subtypes of classical CJD.

In total, 14/15 inoculated 129VV Tg152c recipients were infected following challenge with 129MV vCJD frontal cortex (S2 Table), eight of which developed clinical prion disease with a mean incubation period of 444 days. 7/14 of the infected mice propagated type 5 PrP^Sc^ (Figs 1A and 2A) accompanied by the presence of characteristic irregular, non-florid PrP plaques in the corpus callosum (Fig 1B) that precisely mirrored what would be expected after transmission of prions from 129MM vCJD patient brain to these mice. However, in the other seven infected mice we saw a novel phenotype (Fig 1A). In these mice, non-florid PrP plaque deposition in the corpus callosum (suggesting the propagation of type 5 PrP^Sc^) was accompanied by a dominant stellate type pattern of PrP deposition throughout the brain (Fig 1B) and the appearance of a distinct pattern of spongiosis (S2 Fig), strongly suggesting prominent co-propagation of another prion strain. Remarkably all mice with this novel neuropathological phenotype showed a previously undocumented human PrP^Sc^ molecular strain type on western blots (Fig 2A), that we have designated PrP^Sc^ type 3* (* denoting stellate type neuropathology). PrP^Sc^ type 3* has a proteinase K-resistant fragment size equivalent to that of PrP^Sc^ type 3 seen in classical (sporadic and iatrogenic) CJD (London classification [26]) (Fig 2A) but a novel PrP glycoform ratio (Fig 2B). Most mice (6/7) with this novel phenotype developed clinical prion disease with a mean incubation period of 434 days (Fig 1A). Accordingly, we were intrigued to see whether other regions of 129MV vCJD brain might also transmit the same type 3* stellate phenotype to our transgenic mice.

### Primary transmission of prions from 129MV vCJD patient parietal cortex to transgenic and wild-type mice

**Fig 4:**
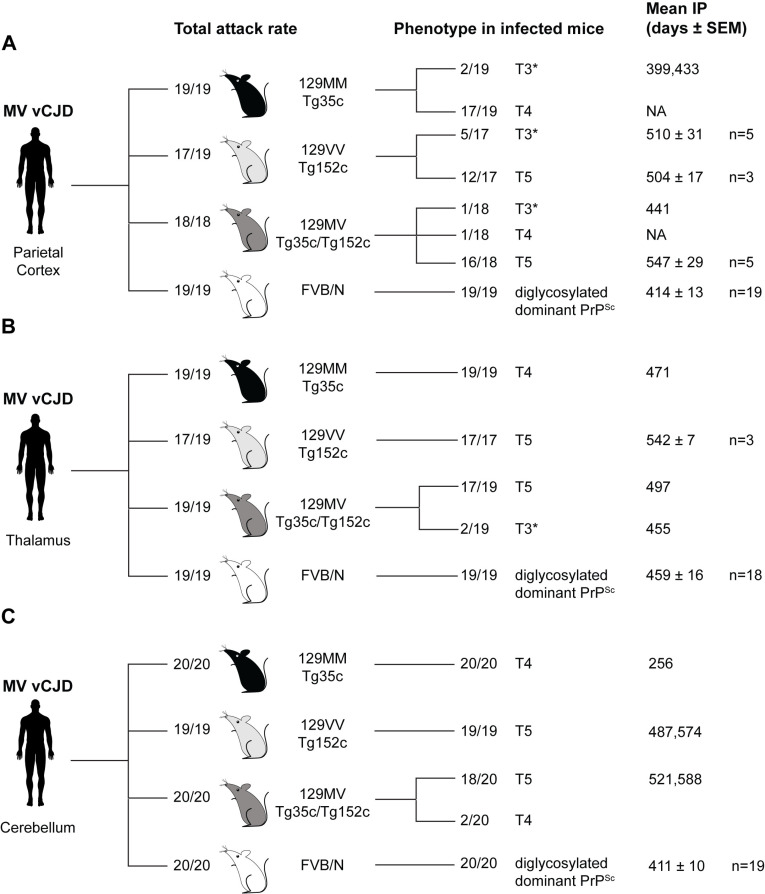
Prion transmission rates and findings in transgenic and wild-type mice challenged with 129MV vCJD patient parietal cortex, thalamus or cerebellum. (A-C) Mice were intracerebrally inoculated with 1% (w/v) homogenate prepared from 129MV vCJD patient parietal cortex (A), thalamus (B) or cerebellum (C). Total attack rate is defined as the total number of clinically affected and subclinically infected mice as a proportion of the number of inoculated mice after post-inoculation periods extending to 600 days. Subclinical prion infection was assessed by immunohistochemical examination of brain for abnormal PrP deposition and western blot analyses of mouse brain homogenates for detectable PrP^Sc^ (by direct western blotting or after NaPTA precipitation if required). The type of PrP^Sc^ (PrP^Sc^ types 4, 5 and 3*; T4, T5, T3*) seen in infected mice is reported together with mean incubation periods (Mean IP) for clinically affected mice in days (where n ≥ 3 the mean ± SEM is reported otherwise individual incubation times are given). NA, not applicable.

Type 4 PrP^Sc^ positive 1% (w/v) parietal cortex homogenate from the 129MV vCJD patient was intracerebrally inoculated into groups of 20 of each mouse line followed by post-inoculation observation periods extending to 600 days. Brains from mice were examined by IHC and biochemical analyses. Findings from these transmissions are summarized in S2 Table and Fig 4A.

Overall, transmissions from the parietal cortex generally mirrored the transmissions seen from the frontal cortex of the MV vCJD patient. Again we observed high levels of sub-clinical infection and low incidence of clinical prion disease in the transgenic mouse lines and a high level of infection and clinical prion disease in FVB/N mice (Fig 4A and S2 Table). With regard to propagated strain types we observed type 4 PrP^Sc^ and typical vCJD neuropathology in the majority (17/19) of infected 129MM Tg35c mice and type 5 PrP^Sc^ and characteristic associated neuropathology in the majority of infected 129VV Tg152c mice (12/17). The majority of 129MV Tg35c/152c mice (16/18 mice) propagated type 5 PrP^Sc^ and one mouse propagated type 4 PrP^Sc^ (Fig 4A). However we also observed the novel type 3* stellate phenotype in 2/19 infected 129MM Tg35c mice, 5/17 infected 129VV Tg152c mice and 1/18 infected 129MV Tg35c/152c mice (Fig 4A). These mice were also all scored clinically sick. The molecular and neuropathological features of the type 3* phenotype were highly similar in mice of all three codon 129 genotypes and congruent with 129VV Tg152c mice challenged with frontal cortex from the 129MV vCJD patient. All infected FVB/N mice (19/19) showed the same characteristic pattern of PrP deposition and diglycosylated PrP dominant PrP^Sc^ type (Fig 4A) to that seen after transmission of frontal cortex from the 129MV vCJD patient to the same mice (Fig 3).

### Primary transmission of prions from 129MV vCJD patient thalamus to transgenic and wild-type mice

Type 4 PrP^Sc^ positive 1% (w/v) thalamus homogenate from the 129MV vCJD patient was intracerebrally inoculated into groups of 20 of each mouse line followed by post-inoculation observation periods extending to 600 days. Brains from mice were examined by IHC and biochemical analyses. Findings from these transmissions are summarized in S2 Table and Fig 4B.

Again in these transmissions we observed high levels of sub-clinical infection and low incidence of clinical prion disease in the transgenic mouse lines and a high level of infection and clinical prion disease in FVB/N mice (Fig 4B and S2 Table). With regard to propagated strains we observed type 4 PrP^Sc^ and typical vCJD neuropathology in all (19/19) infected 129MM Tg35c mice and type 5 PrP^Sc^ and characteristic associated neuropathology in all (17/17) infected 129VV Tg152c mice (Fig 4B). In 129MV Tg35c/152c mice we observed 17/19 mice with type 5 PrP^Sc^ and characteristic associated neuropathology together with 2/19 mice that showed the novel PrP^Sc^ type 3* phenotype (Fig 4B). All infected FVB/N mice (19/19) showed the same characteristic pattern of PrP deposition and diglycosylated PrP dominant PrP^Sc^ type (Fig 4B) as seen after transmission of frontal cortex or parietal cortex from the 129MV vCJD patient to the same mice.

### Primary transmission of prions from 129MV vCJD patient cerebellum to transgenic and wild-type mice

Type 4 PrP^Sc^ positive 1% (w/v) cerebellum homogenate from the 129MV vCJD patient was intracerebrally inoculated into groups of 20 of each mouse line followed by post-inoculation observation periods extending to 600 days. Brains from mice were examined by IHC and biochemical analyses. Findings from these transmissions are summarized in S2 Table and Fig 4C.

As with transmissions from other brain regions we observed high levels of sub-clinical infection and low incidence of clinical prion disease in the transgenic mouse lines and a high level of infection and clinical prion disease in FVB/N mice (Fig 4C and S2 Table). With regard to propagated strains we observed type 4 PrP^Sc^ and typical vCJD neuropathology in all (20/20) 129MM Tg35c mice and type 5 PrP^Sc^ and characteristic associated neuropathology in all (19/19) recipient 129VV Tg152c mice (Fig 4C). In 129MV Tg35c/152c mice, 18/20 mice propagated type 5 PrP^Sc^ with characteristic associated neuropathology and 2/20 mice had a vCJD phenotype with propagation of type 4 PrP^Sc^ and the occurrence of occasional florid plaques in the cortex (Fig 4C). In distinction to transmissions from frontal cortex, parietal cortex and thalamus, no transgenic mouse challenged with 129MV vCJD cerebellum showed the novel PrP^Sc^ type 3* phenotype. All infected FVB/N mice (20/20) challenged with cerebellum showed the same characteristic pattern of PrP deposition and diglycosylated PrP dominant PrP^Sc^ type (Fig 4C) as seen in transmissions of frontal cortex, parietal cortex and thalamus from the 129MV vCJD patient to the same mice.

### Summary of primary transmissions of 129MV vCJD brain regions to transgenic and wild-type mice

Collective findings from transmissions of all four brain regions from the 129MV vCJD patient indicate that the vCJD prion strain (type 4 PrP^Sc^ propagated on 129M PrP) is the dominant prion strain in the patient’s brain. However a novel prion strain appears to be present at lower levels in the frontal cortex, parietal cortex and thalamus. This previously undocumented human prion strain was amplified in a proportion of transgenic mice of all three *PRNP* codon 129 genotypes and showed a common molecular and neuropathological phenotype characterized by propagation of PrP^Sc^ type 3* and a novel stellate type pattern of PrP deposition throughout the brain. This novel PrP^Sc^ type was also associated with a much higher incidence of clinical disease with 88% of mice with PrP^Sc^ type 3* being clinically affected. This is a much higher rate than is seen with the other PrP^Sc^ types generated in transgenic mice in this study.

### Secondary transmissions of prions from transgenic mouse brain

Four transgenic mouse brains from the primary transmissions of 129MV vCJD frontal cortex were selected for secondary transmission. These comprised: a 129MM Tg35c mouse propagating PrP^Sc^ type 4 with abundant florid plaques; a 129VV Tg152c mouse propagating PrP^Sc^ type 3* with novel stellate type neuropathology; a 129VV Tg152c mouse propagating PrP^Sc^ type 5 with characteristic associated PrP plaques in the corpus callosum: a 129MV Tg35c/152c mouse propagating PrP^Sc^ type 5 with characteristic associated PrP plaques in the corpus callosum. Figs 1 and 2 respectively, show the PrP deposition patterns and PrP^Sc^ types in these brains. 1% (w/v) homogenates from these brains were intracerebrally inoculated into groups of 20 of the same transgenic mouse lines or wild-type FVB/N mice that were used for the primary transmission series. Mice were observed over post-inoculation periods extending to 600 days and brains were collected and examined by IHC and biochemical analyses. Transmission data are reported in S3 Table.

### Transmission of 129MM Tg35c mouse brain with PrP^Sc^ type 4 and abundant florid PrP plaques

**Fig 5:**
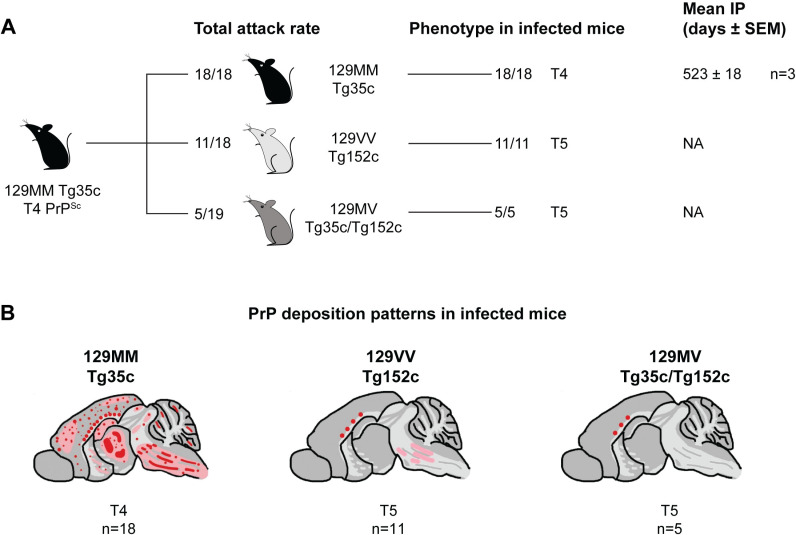
Prion transmission rates and findings in transgenic mice challenged with 129MM Tg35c-passaged 129MV vCJD patient frontal cortex. (A) Overview of transmission rates and molecular and neuropathological findings in recipient mice. Mice were intracerebrally inoculated with 1% (w/v) homogenate prepared from a 129MM Tg35c mouse brain propagating type 4 PrP^Sc^ derived from primary transmission of 129MV vCJD patient frontal cortex (shown in Figs 1 and 2). Total attack rate is defined as the total number of clinically affected and subclinically infected mice as a proportion of the number of inoculated mice after post-inoculation periods extending to 600 days. Subclinical prion infection was assessed by immunohistochemical examination of brain for abnormal PrP deposition. The phenotype in infected mice (T4, T5) is reported together with mean incubation period (Mean IP) for clinically affected mice in days (where **n** ≥ 3 the mean ± SEM is reported). NA, not applicable. (B) PrP deposition patterns in infected transgenic mouse brains by IHC using anti-PrP monoclonal antibody 3F4. Panels show schematic drawings in which the overall spatial distribution and intensity of abnormal PrP deposition is indicated by red shading with PrP plaques shown as red dots.

**Fig 6:**
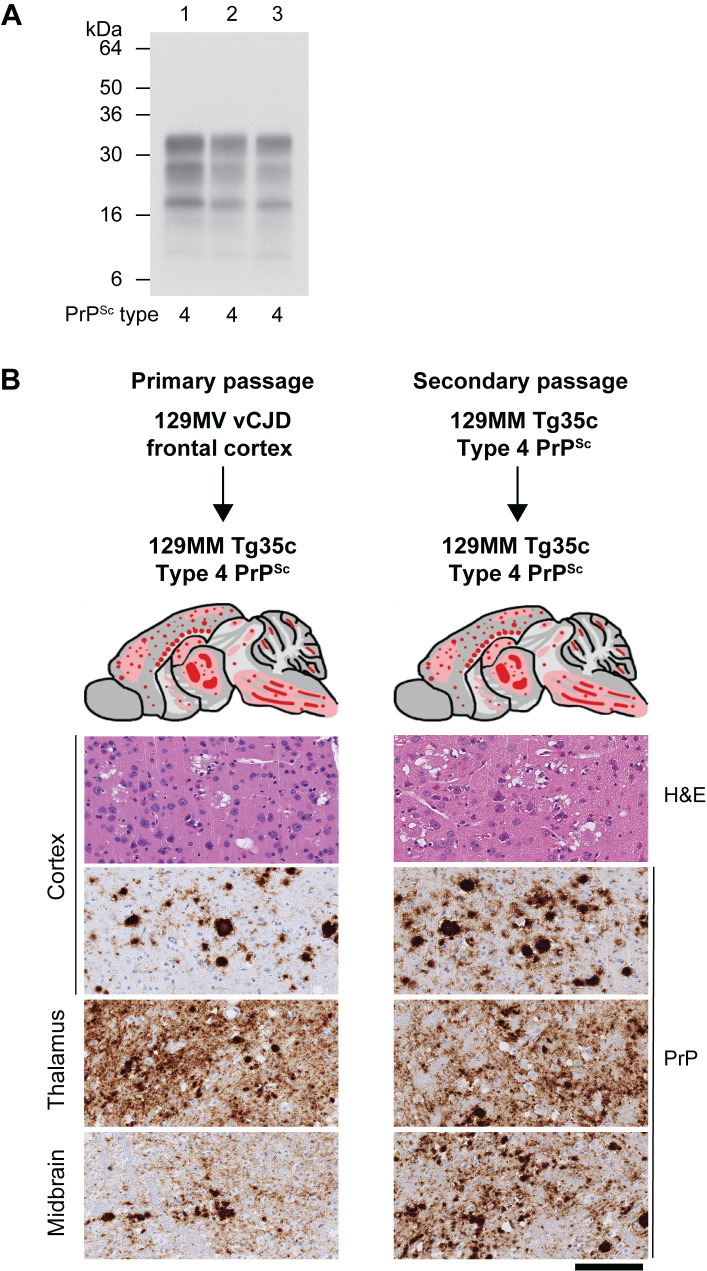
Primary and secondary transmission of 129MV vCJD patient frontal cortex in 129MM Tg35c mice. (A) Western blot of proteinase K-digested 10% (w/v) brain homogenates using anti-PrP monoclonal antibody 3F4 and high sensitivity enhanced chemiluminescence. The type of PrP^Sc^ seen in infected brain is reported below each lane. Lane 1, reference case of 129MM vCJD patient frontal cortex, lane 2, 129MM Tg35c mouse brain from primary transmission of 129MV vCJD patient frontal cortex, lane 3, 129MM Tg35c mouse brain challenged with 129MM Tg35c-passaged 129MV vCJD patient frontal cortex. (B) PrP deposition patterns in infected 129MM Tg35c mouse brains by IHC using anti-PrP monoclonal antibody 3F4. Upper panels show schematic drawings in which the overall spatial distribution and intensity of abnormal PrP deposition is indicated by red shading with PrP plaques shown as red dots. Lower panels show representative haematoxylin- and eosin-stained sections (H&E) showing spongiform neurodegeneration including florid plaques in the cortex and representative PrP IHC (PrP) with anti-PrP monoclonal antibody 3F4 in cortex, thalamus and midbrain. Scale bar, all lower panels, 100 µm.

Transmissions of this isolate were entirely consistent with those of type 4 PrP^Sc^ from 129MM Tg35 mice generated after challenge with 129MM vCJD patient brain. All 129MM Tg35c recipient mice were infected (18/18) but only 3 developed clinical prion disease with a mean incubation period of 523 days (S3 Table and Fig 5A). Infected 129MM Tg35c mice propagated type 4 PrP^Sc^ (Fig 6A) accompanied by the occurrence of florid PrP plaques (Fig 6B). In 129VV Tg152c mice and 129MV Tg35c/Tg152c mice, 11/18 and 5/19 inoculated recipients respectively were infected; none of the infected mice developed clinical prion disease (S3 Table and Fig 5A). Infected mice showed the presence of non-florid PrP plaques in the corpus callosum (Fig 5B), consistent with the propagation of type 5 PrP^Sc^.

### Transmission of 129VV Tg152c mouse brain with PrP^Sc^ type 3* and novel stellate type PrP neuropathology

**Fig 7:**
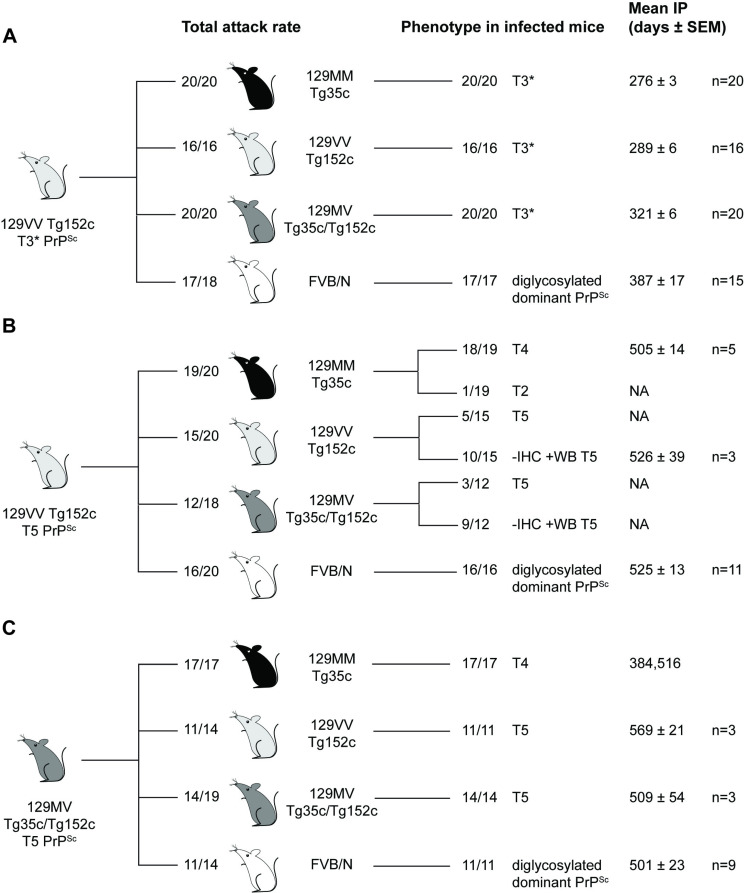
Summary of secondary prion transmissions in transgenic and wild-type mice. (A-C) Mice were intracerebrally inoculated with 1% (w/v) brain homogenate prepared from prion-infected transgenic mice challenged with 129MV vCJD patient frontal cortex. Patterns of abnormal PrP deposition by IHC and PrP^Sc^ types in the infected mice from which the inocula were derived are shown in Figs 1B and 2A, respectively. Total attack rate is defined as the total number of clinically affected and subclinically infected mice as a proportion of the number of inoculated mice after post-inoculation periods extending to 600 days. Subclinical prion infection was assessed by immunohistochemical examination of brain for abnormal PrP deposition and western blot analyses of mouse brain homogenates for detectable PrP^Sc^ (by direct western blotting or after NaPTA precipitation if required). The type of PrP^Sc^ (PrP^Sc^ types 2, 4, 5 and 3*, T2, T4, T5, T3*) seen in infected mice is reported together with mean incubation periods (Mean IP) for clinically affected mice in days (where n ≥ 3 the mean ± SEM is reported otherwise individual incubation times are given). NA, not applicable.

**Fig 8:**
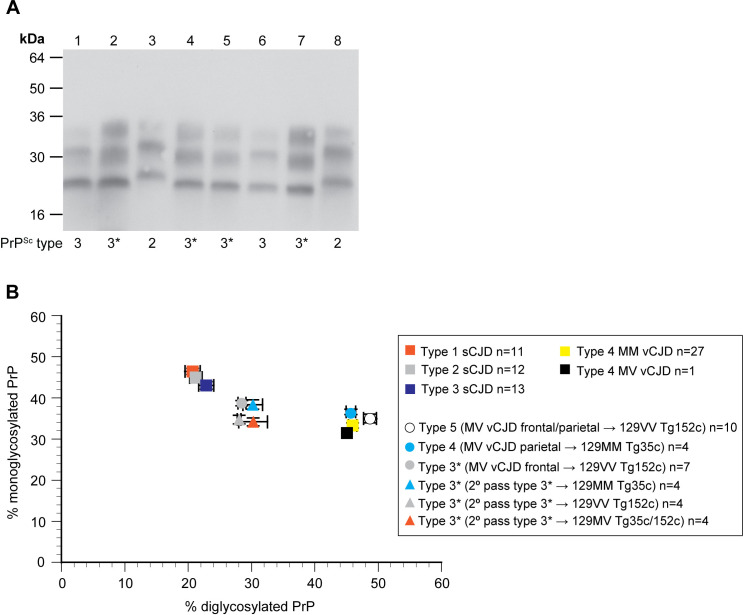
PrP^Sc^ types and glycoform ratios in transgenic mice from secondary transmissions. (A) Western blot of proteinase K-digested 10% (w/v) brain homogenates from transgenic mice or human reference cases of sporadic CJD (sCJD) (PrP^Sc^ type 2 129MM and type 3 129MV) using anti-PrP monoclonal antibody 3F4 and high sensitivity enhanced chemiluminescence. The propagated PrP^Sc^ type (PrP^Sc^ types 2, 3, 3*) is shown below each lane. Lanes 1, 3, 6 and 8, sCJD brain. Lane 2, PrP^Sc^ type 3*-positive 129VV Tg152c brain from primary transmission of 129MV vCJD patient frontal cortex. Lanes 4, 5 and 7, secondary transmissions of PrP^Sc^ type 3*-positive 129VV Tg152c brain to further transgenic mice; lane 4, 129MM Tg35c brain, lane 5, 129VV Tg152c brain, lane 7, 129MV Tg35c/Tg152c brain. (B) Ratios of the di- and mono-glycosylated protease-resistant PrP glycoforms seen in PrP^Sc^ from the brains of transgenic mice, 129MV vCJD patient frontal cortex, 129MM vCJD patient frontal cortex or reference cases of sCJD patient frontal cortex with PrP^Sc^ types 1, 2 or 3 (London classification, [26]). Where sample size is ≥ 3 symbols show mean percentage ± SEM otherwise individual samples are shown. In some cases the error bars are smaller than the symbols used. Squares, human samples, circles, mouse primary transmission samples, triangles, mouse secondary transmission samples.

**Fig 9:**
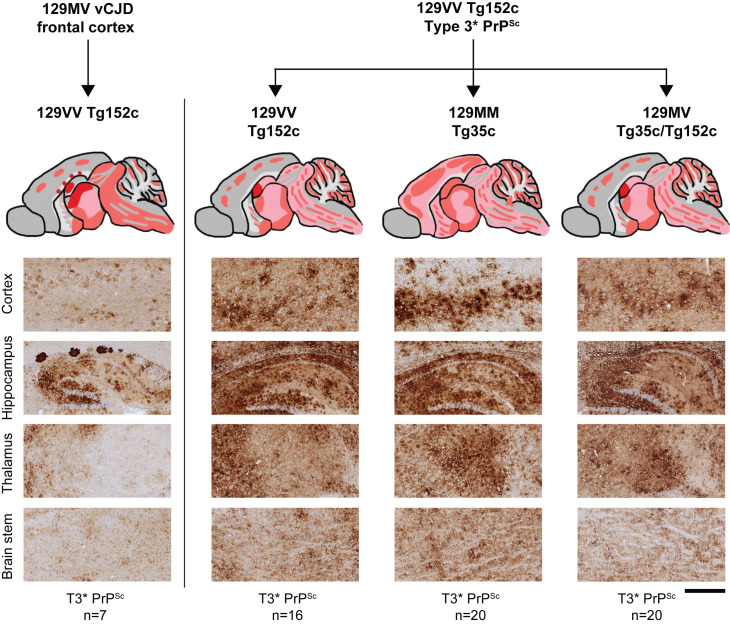
Abnormal PrP deposition in the brain of transgenic mice propagating type 3* PrP^Sc^. The provenance of the brain sample is designated above each column and the type of PrP^Sc^ (PrP^Sc^ type 3*; T3*) seen in infected brain is designated below. Upper panels show schematic drawings in which the overall spatial distribution and intensity of abnormal PrP deposition is indicated by red shading with PrP plaques shown as red dots. Lower panels show representative PrP IHC in cortex, hippocampus (including corpus callosum), thalamus and brain stem using anti-PrP monoclonal antibody 3F4. Scale bar, 150 µm for row 1 (cortex) and 300 µm for rows 2 (hippocampus), 3 (thalamus), and 4 (brain stem).

Transmissions from this isolate were remarkable. We observed 100% attack rates of both infection and clinical prion disease in all three transgenic mouse lines with mean incubation periods of around 300 days (S3 Table and Fig 7A). Affected transgenic mice of all three codon 129 genotypes propagated PrP^Sc^ type 3* (Figs 7A, 8A and 8B) accompanied by abundant stellate type PrP neuropathology throughout the brain (Fig 9). Detailed overviews of the patterns of PrP deposition and spongiosis in these mice are provided in S3 Fig.

In FVB/N mice we also observed high rates of infection (17/18 mice) and incidence of clinical prion disease (15/18 mice) with a mean incubation period of 387 days (S3 Table and Fig 7A). The neuropathology and diglycosylated PrP dominant PrP^Sc^ type propagated in this transmission was indistinguishable from that seen in infected FVB/N mice from the primary transmissions of 129MV vCJD patient brain regions. These findings suggest that co-propagating type 5 PrP^Sc^ in the 129VV Tg152c brain inoculum is likely to be the infecting prion strain in FVB/N mice and that PrP^Sc^ type 3* in the inoculum may not seed wild-type mouse PrP to produce a different neuropathological phenotype. However further serial transmissions will be required to verify this.

### Transmission of 129VV Tg152c mouse brain with type 5 PrP^Sc^ and PrP plaques in the corpus callosum

The transmission properties of 129VV Tg152c mouse brain with type 5 PrP^Sc^ and PrP plaques in the corpus callosum was entirely consistent with the previously documented transmission properties of type 5 PrP^Sc^ isolates from 129VV Tg152 mice produced after challenge with 129MM vCJD patient brain [8,9]. In 129MM Tg35c mice 19/20 mice were infected but only five developed clinical prion disease with a mean incubation period of 505 days (S3 Table and Fig 7B). 18/19 of the infected mice propagated type 4 PrP^Sc^ which in the majority of mice was accompanied by the occurrence of florid PrP plaques (Fig 7B). 1/19 mice propagated a sporadic CJD-like type 2 PrP^Sc^ molecular strain type accompanied by diffuse PrP deposition (Fig 7B). The behaviour of the 129VV Tg152c type 5 PrP^Sc^ isolate generated after challenge with 129MV vCJD patient frontal cortex therefore precisely mirrors our previous findings with type 5 PrP^Sc^ generated in 129VV Tg152 mice challenged with 129MM vCJD patient brain [8]. In keeping with these findings, transmission of the type 5 PrP^Sc^ isolate in both 129VV Tg152c mice and 129MV Tg35c/152c mice produced lower overall rates of infection and incidence of clinical prion disease (S3 Table and Fig 7B). In 129VV Tg152 mice 15/20 mice were infected but only 3 mice developed clinical prion disease with a mean incubation period of 526 days (S3 Table and Fig 7B). All infected mice propagated type 5 PrP^Sc^, however only 5 mice were scored positive by IHC with observation of occasional non-florid PrP plaques (Fig 7B). These data are congruent with our previous findings which similarly showed a lack of adaptation of the type 5 PrP^Sc^ prion strain when serially passed in 129VV Tg152 mice [8]. In 129MV Tg35c/152c mice challenged with the type 5 PrP^Sc^ isolate, 12/18 mice were infected however none developed clinical prion disease (Fig 7B). All infected mice propagated type 5 PrP^Sc^ however only 3 mice were scored positive by IHC with occurrence of occasional non-florid PrP plaques in the corpus callosum (Fig 7B). Again, these findings are consistent with previous transmissions of type 5 PrP^Sc^ isolates in 129MV transgenic mice [9]. In FVB/N mice challenged with the type 5 PrP^Sc^ 129VV Tg152c mouse brain isolate 16/20 mice were infected of which 11 developed clinical prion disease with a mean incubation period of 525 days (S3 Table and Fig 7B). All infected mice showed the same characteristic neuropathology and diglycosylated PrP dominant PrP^Sc^ type (Fig 7B) as seen in previous transmissions of type 5 PrP^Sc^ to FVB/N mice [8] and congruent with transmissions of 129MM or 129MV vCJD patient brain samples to these mice.

### Transmission of 129MV Tg35c/Tg152c mouse brain with type 5 PrP^Sc^ and PrP plaques in the corpus callosum

Secondary transmission of this isolate essentially mirrored those described above for the type 5 PrP^Sc^ 129VV Tg152c mouse brain isolate. Details of these transmissions are provided in S3 Table and Fig 7C and do not require further elaboration.

### Summary of secondary transmissions in transgenic and wild-type mice

Transmissions of the type 4 PrP^Sc^ isolate from 129MM Tg35c mouse brain were entirely consistent with previous serial transmission studies of type 4 PrP^Sc^ isolates originating from 129MM vCJD patient brain. However, transmissions of the 129VV Tg152c mouse brain propagating type 3* PrP^Sc^ with novel stellate type PrP neuropathology were remarkable and resulted in uniform occurrence of infection and clinical prion disease in all inoculated transgenic mice regardless of *PRNP* codon 129 genotype. All affected mice showed faithful maintenance of the novel type 3* PrP^Sc^ molecular strain type accompanied by a prominent stellate pattern of PrP deposition. Transmissions of type 5 PrP^Sc^ isolates from 129VV Tg152c and 129MV Tg35c/152c mouse brains were entirely consistent with those of type 5 PrP^Sc^ isolates generated previously in 129VV Tg152 mice challenged with 129MM vCJD patient brain.

### Analyses of 129MV vCJD peripheral tissues for detectable PrP^Sc^

Peripheral tissues available from the 129MV vCJD patient comprised appendix, mesenteric lymph nodes and spleen. We prepared 10% (w/v) D-PBS homogenates from ~300 mg tissue samples using sterile glass tissue grinders. In total we prepared twenty-two homogenates, two from appendix, two from mesenteric lymph nodes and eighteen from spleen. 500 µl aliquots of each 10% (w/v) homogenate were analysed by sodium phosphotungstic acid (NaPTA) precipitation and high sensitivity western blotting for detectable PrP^Sc^ using established procedures [33–35]. This method facilitates highly efficient recovery and detection of PrP^Sc^ from 129MM vCJD peripheral tissue homogenates when present at levels 10^4^–10^5^-fold lower than in 129MM vCJD brain [24,31,33,36–39].

All peripheral tissue homogenates from the 129MV vCJD patient were devoid of detectable PrP^Sc^ following NaPTA precipitation with the exception of one homogenate prepared from appendix which showed PrP^Sc^ close to the detection limit of the method (S4A Fig). We estimated that the PrP^Sc^ level in this 10% (w/v) appendix homogenate was about 10^4^ fold-lower than that present in 10% (w/v) homogenate prepared from the 129MV vCJD patient’s frontal cortex. These biochemical findings are consistent with our original IHC findings where we found that spleen contained only minute amounts of abnormal PrP deposition [18].

### Primary transmissions of 129MV vCJD peripheral tissues to transgenic and wild-type mice

**Fig 10:**
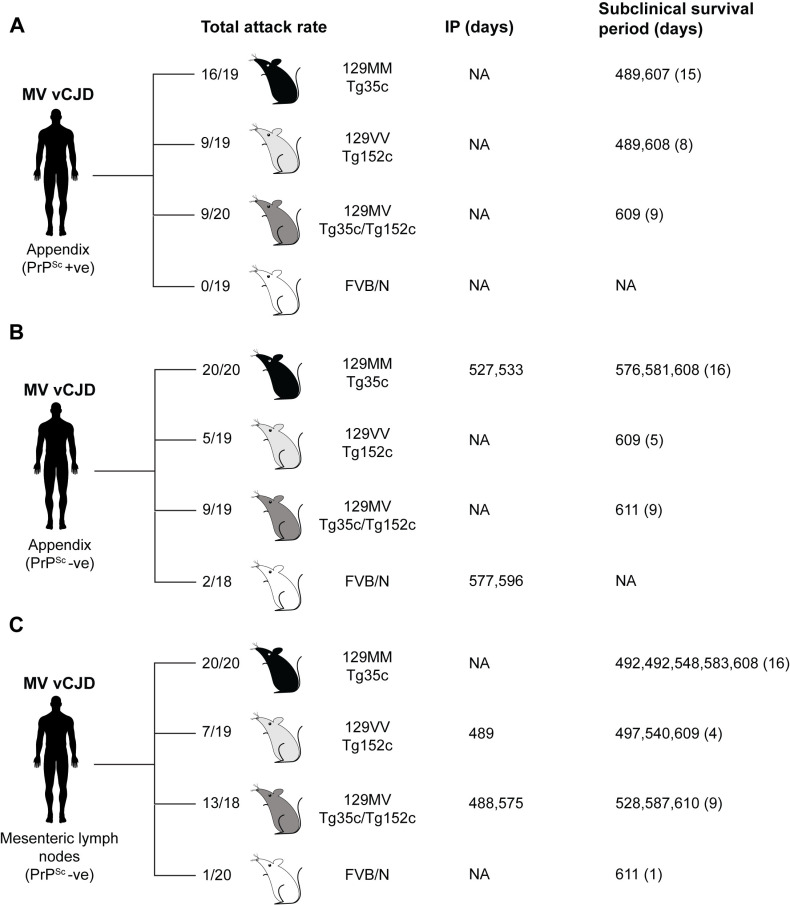
Prion transmission rates and findings in transgenic and wild-type mice challenged with 129MV vCJD patient appendix and mesenteric lymph node. Mice were intracerebrally inoculated with 1% (w/v) homogenate prepared from 129MV vCJD patient appendix (A and B), and mesenteric lymph nodes (C). The status of whether PrP^Sc^ was detectable in the homogenate is shown. Total attack rate is defined as the total number of clinically affected and subclinically infected mice as a proportion of the number of inoculated mice after post-inoculation periods extending to 600 days. Subclinical prion infection was assessed by immunohistochemical examination of brain for abnormal PrP deposition and western blot analyses of mouse brain homogenates for detectable PrP^Sc^ after NaPTA precipitation. Incubation periods (IP) for individual clinically affected mice are reported. Survival periods of subclinically infected mice reports the number of days between inoculation and culling due to inter-current illness or termination of the experiment. The survival periods of individual mice culled before termination of the experiment are shown together with the survival period at termination of the experiment, with number of mice culled at this point shown in parentheses. NA, not applicable.

Six peripheral tissue homogenates were used for primary transmissions. These comprised, one PrP^Sc^-positive appendix homogenate, one PrP^Sc^-negative appendix homogenate, one PrP^Sc^-negative mesenteric lymph node pooled homogenate (pool of two homogenates) and three PrP^Sc^-negative spleen homogenate pools designated spleen pool A (prepared from spleen homogenates 1-6), spleen pool B (prepared from spleen homogenates 7-12) and spleen pool C (prepared from spleen homogenates 13-18). 1% (w/v) homogenates were intracerebrally inoculated into groups of 20 of the same mouse lines that were used for the primary transmission series of 129MV vCJD patient brain. Mice were observed over post-inoculation observation periods extending to 600 days. Brains from recipient mice were examined by IHC and biochemical analyses. Findings are summarized in S4 and S5 Tables and Fig 10A–C.

### Primary transmissions of 129MV vCJD patient appendix to transgenic and wild-type mice

**Fig 11:**
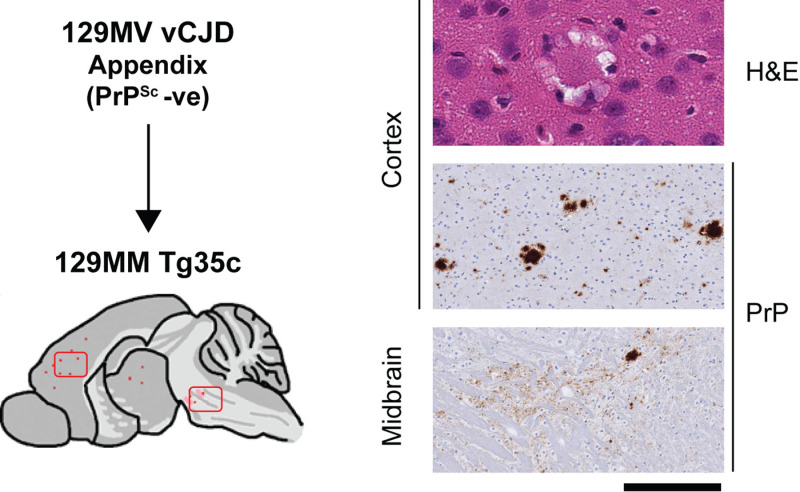
Abnormal PrP deposition in the brain of 129MM Tg35c mice after primary transmission of 129MV vCJD patient appendix. Mice were intracerebrally inoculated with 1% (w/v) homogenate prepared from 129MV vCJD patient appendix homogenate (negative for detectable PrP^Sc^). The schematic drawing shows the spatial distribution of PrP plaques detected by IHC with anti-PrP monoclonal antibody 3F4 as red dots. Brain sections from areas defined by rectangles in the schematic drawing are shown on the right; representative haematoxylin- and eosin-stained sections (H&E) showing spongiform neurodegeneration and florid plaques in the cortex and representative PrP IHC (PrP) in cortex and mid brain. Scale bar, H&E 50 µm, PrP 200 µm.

Both PrP^Sc^-positive and PrP^Sc^-negative appendix homogenates transmitted high attack rates of infection in 129MM Tg35c mice (16/19 and 20/20 of recipient mice, respectively) however only two infected mice developed clinical prion disease (S4 Table and Fig 10A and 10B). In these transmissions western blot detection of PrP^Sc^ required NaPTA precipitation of 250 µl 10% (w/v) mouse brain homogenate (S4 Fig) and homogenates that were most strongly scored positive for PrP^Sc^ after NaPTA precipitation were scored negative when 20 µl aliquots of 10% (w/v) homogenates were analysed by direct western blotting. All infected 129MM Tg35c mice showed propagation of diglycosylated PrP dominant PrP^Sc^ consistent with propagation of type 4 PrP^Sc^ in these mice (S4B and S4C Fig). The requirement for NaPTA precipitation for detection of PrP^Sc^ indicates low levels of propagating prions in the brain of recipient 129MM Tg35c mice, which in turn reflects low prion titres in the 129MV patient’s appendix. Consistent with these biochemical findings, low levels of PrP deposition were detected by IHC and consisted of infrequent, small PrP plaques in cortex and midbrain, some of which were florid (Fig 11). The morphologies and distribution of the florid PrP plaques in the cortex and midbrain were consistent with the early stages of prion infection seen in 129MM Tg35c mice following challenge with prions from 129MM vCJD patient brain or in 129MM Tg45 mice following challenge with prions from 129MM vCJD patient appendix [24].

In 129VV Tg152c and 129MV Tg35c/152c mice, both PrP^Sc^-positive and PrP^Sc^-negative appendix homogenates transmitted lower rates of infection with no incidence of clinical prion disease (S4 Table and Fig 10A and 10B). NaPTA precipitation analyses of recipient brain homogenate was again required for PrP^Sc^ detection and showed the propagation of diglycosylated PrP dominant PrP^Sc^ in all infected mice, consistent with propagation of type 5 PrP^Sc^ in these mice. Concordant with low levels of detectable PrP^Sc^, very low levels of PrP deposition were seen by IHC, which when detected, comprised small non-florid plaques in the corpus callosum, again consistent with propagation of type 5 PrP^Sc^.

In wild-type FVB/N mice, inoculation of PrP^Sc^-positive and PrP^Sc^-negative appendix homogenate resulted in low infection rates of 0/19 and 2/18 inoculated recipients respectively (S4 Table and Fig 10A and 10B). Both infected mice developed clinical prion disease (S4 Table and Fig 10B) and propagated diglycosylated PrP dominant PrP^Sc^ (detectable by direct western blotting) and patterns of PrP deposition by IHC mirroring that seen after transmission of either 129MM vCJD or 129MV vCJD patient brain samples in these mice.

### Primary transmissions of 129MV vCJD patient mesenteric lymph node to transgenic and wild-type mice

Pooled PrP^Sc^-negative mesenteric lymph node homogenate transmitted a high attack rate of infection in 129MM Tg35c mice (20/20 of recipient mice) however none of the infected mice developed clinical prion disease (S4 Table and Fig 10C). As seen with transmissions of patient appendix, western blot detection of PrP^Sc^ required NaPTA precipitation of 250 µl 10% (w/v) mouse brain homogenate. All infected mice showed propagation of diglycosylated PrP dominant PrP^Sc^ consistent with propagation of type 4 PrP^Sc^ and IHC showed infrequent, small PrP plaques in cortex and midbrain, some of which were florid. In 129VV Tg152c mice challenged with mesenteric lymph node 7/19 mice brain showed evidence of infection (S4 Table and Fig 10C) after NaPTA precipitation of 250 µl 10% (w/v) brain homogenate with one mouse developing clinical prion disease. All infected mice showed propagation of diglycosylated PrP dominant PrP^Sc^ consistent with propagation of type 5 PrP^Sc^, however all mice were scored negative for abnormal PrP deposition by IHC. In 129MV Tg35c/Tg152c mice a higher rate of infection was observed and two mice developed clinical prion disease. 13/18 brains were scored positive for PrP^Sc^ after NaPTA precipitation of 250 µl 10% (w/v) brain homogenate (S4 Table and Fig 10C). Propagation of diglycosylated PrP dominant PrP^Sc^ was seen in all infected recipients consistent with propagation of either type 4 or type 5 PrP^Sc^ in these mice. IHC analyses of brain showed abnormal PrP deposition in only a single brain which comprised a single large non-florid PrP plaque in the corpus callosum, consistent with propagation of type 5 PrP^Sc^. In wild-type FVB/N mice a low infection rate of 1/20 mice was seen after challenge with mesenteric lymph node homogenate (S4 Table and Fig 10C). This animal was subclinically infected and had a pattern of PrP deposition by IHC consistent with that seen in the early stages of transmission of 129MM vCJD patient brain samples to these mice.

### Primary transmissions of 129MV vCJD patient spleen to transgenic and wild-type mice

Transmissions of PrP^Sc^-negative spleen homogenate pools A, B and C to 129MM Tg35c mice resulted in asymptomatic infection of only a single mouse (from spleen homogenate pool B) (S5 Table). The single infected brain showed weak propagation of diglycosylated PrP dominant PrP^Sc^ (that required NaPTA precipitation of 250 µl 10% (w/v) brain homogenate for detection) and the presence of two PrP microplaques in the cortex and midbrain by IHC (S5 Table). Based upon the extremely poor transmission rate of 129MV patient spleen in 129MM Tg35c mice, and the recognition that appendix and mesenteric lymph node showed the highest infection rates in these mice, brains from the other lines of mice challenged with spleen were not analysed.

### Summary of primary transmissions of 129MV vCJD peripheral tissues to transgenic and wild-type mice

Transmissions of 129MV vCJD patient appendix, mesenteric lymph node and spleen in transgenic and wild-type mice indicate low titres of prions in these tissues. The transmission properties and the molecular and neuropathological findings in infected mice are all consistent with transmission of the vCJD prion strain. These transmissions showed no evidence for the emergence of the novel type 3* prion strain from the patient’s lymphoid tissues.

## Discussion

Generous consent for research from the patient and their family has provided the opportunity to study the distribution and strain properties of prions propagated in the brain and lymphoid tissues of the first neuropathologically confirmed case of vCJD in a codon 129 heterozygous individual [18].

The vCJD prion strain (type 4 PrP^Sc^ propagated on human PrP 129M) is clearly the dominant prion strain in the 129MV vCJD patient’s brain and at primary transmission the majority of recipient humanized transgenic mice showed phenotypes that were entirely consistent with those from our historical transmission series of numerous 129MM vCJD patient brain samples. However in sharp distinction to findings with 129MM vCJD patient brain samples, the 129MV vCJD patient’s brain tissue was found to harbour a previously unrecognised human prion strain, that we have designated type 3*. Type 3* prions emerged in recipient transgenic mice after primary challenge with three distinct 129MV vCJD patient brain regions, and after secondary passage from transgenic mouse brain, type 3* prions were uniformly lethal in recipient transgenic mice of all human codon 129 genotypes with similar short mean incubation periods. The type 3* prion strain appears restricted to propagating on human PrP, as primary transmissions of 129MV vCJD brain tissue in wild-type mice produced a single phenotype mirroring that seen in previous transmissions of numerous 129MM vCJD brain samples to the same wild-type mice. These findings in FVB/N mice are therefore consistent with findings from an earlier transmission study of 129MV vCJD brain tissue in wild-type RIII mice [40]. While caution must be exercised when extrapolating findings from a single 129MV vCJD patient, this first case demonstrates that 129MV vCJD is distinct from 129MM vCJD as we were able to isolate a novel human prion strain from the patient’s brain tissue. Whether co-propagation of the novel type 3* prion strain would be common to all 129MV vCJD cases can only be addressed if further cases of 129MV vCJD are identified.

Depending upon the density of lymphoid follicles, PrP^Sc^ concentrations in 129MM vCJD patient peripheral tissues can vary enormously, with levels relative to brain as high as 10% in tonsil or as low as 0.002% in rectum [24,31,33,36–39,41]. In distinction to type 4 PrP^Sc^ found in vCJD brain [26,27], lymphoreticular tissues in 129MM vCJD patients propagate a different PrP^Sc^ glycotype (type 4t) [33,41], however the prion strain transmission properties of type 4t PrP^Sc^ and type 4 PrP^Sc^ appear to be congruent in our transgenic mice [24,31].

On the basis of detectable levels of PrP^Sc^, the 129MV vCJD patient’s lymphoreticular tissues have a level of prion colonisation that is lower than that seen in most 129MM vCJD patients [24,31,33,36–39,41]. The demonstration of weak PrP^Sc^**-**positivity in 129MV vCJD appendix does however suggest that subclinically infected 129MV individuals may have been detected by anonymous screening of surgical appendix tissue [19–21]. The phenotypes seen in transmissions of peripheral lymphoid tissues from the 129MV vCJD patient in both humanized transgenic mice and wild-type mice were consistent with previous transmissions of lymphoid tissues from 129MM vCJD patients [24,31]. Notably, the emergence of the novel type 3* prion strain was not seen following primary transmission of 129MV vCJD patient lymphoid tissue. These data suggest that BSE prion-infected asymptomatic 129MV vCJD carriers are likely to pose similar risks for iatrogenic transmission of human prions from peripheral tissues to those already recognized for asymptomatic 129MM vCJD carriers.

## Materials and methods

### Ethics statement

All work with prion-infected samples was conducted in microbiological containment level 3 facilities with strict adherence to safety protocols. Storage and biochemical analyses of post-mortem human tissue samples and transmission studies to mice were performed with written informed consent from patients with capacity to give consent. Where patients were unable to give informed consent, written assent was obtained from their relatives in accordance with UK legislation and Codes of Practice. Samples were stored and used in accordance with the Human Tissue Authority Codes of Practice and in line with the requirements of the Human Tissue Authority licence held by UCL Queen Square Institute of Neurology. All work with human tissue was performed with approval from the National Hospital for Neurology and Neurosurgery and the University College London Institute of Neurology Joint Research Ethics Committee (now National Research Ethics Service Committee, London – Queen Square) - REC references: 03/N036, 03/N038 and 03/N133. Work with mice was performed under approval and licence granted by the UK Home Office (Animals (Scientific Procedures) Act 1986); Project Licence numbers 70/9022 and PP3260075 and conformed to UCL institutional and ARRIVE guidelines (www.nc3rs.org.uk/ARRIVE/). All protocols and procedures were approved by the MRC Prion Unit at University College London Animal Welfare and Ethical Review Body.

### Preparation of tissue homogenates and western blotting

Tissue samples were prepared as 10% (w/v) homogenates in Dulbecco’s sterile phosphate buffered saline lacking Ca^2+^ and Mg^2+^ ions (D-PBS) using Duall glass tissue grinders (Anachem Ltd) or a Precellys Evolution tissue homogenizer (Bertin Instruments). Proteinase K digestion (50 or 100 μg/ml final protease concentration in the sample, 1h, 37 °C), electrophoresis and immunoblotting was performed as described previously [34,35]. Samples were analysed on Novex 16% Tris-Glycine gels (Thermo Fisher Scientific) or 15% Criterion Tris-HCl gels (Bio-Rad Laboratories) and calibrated using the Seeblue Pre-stained Protein Standard from Invitrogen (Thermo Fisher Scientific). High sensitivity immunoblot detection of human or mouse PrP was performed using anti-PrP monoclonal antibodies 3F4 (epitope spanning residues 104-113 of human PrP) (BioLegend UK Ltd) or 6D11 (epitope spanning residues 93-109 of mouse PrP) (BioLegend UK Ltd), respectively. For analysis of PrP glycoforms, blots were developed in chemifluorescent substrate (AttoPhos; Promega) and visualized on a Storm 840 phosphoimager (Amersham) using ImageQuaNT software (Amersham) [34,35]. Sodium phosphotungstic acid (NaPTA) precipitation of PrP^Sc^ (from 500 µl 10% (w/v) tissue homogenates from the 129MV patient or 250 µl 10% (w/v) mouse brain homogenates) was performed as described previously [33–35].

### Transmission studies

Transmission studies to mice were performed in the Unit’s microbiological containment level 3 Biological Services Facility with strict adherence to safety protocols. We performed 10 primary transmissions of tissue homogenates from the 129MV patient’s brain and lymphoreticular tissues into our highly characterized lines of transgenic mice that are fully susceptible to human prions [6–10,23–25] and wild type mice. Inocula from patient tissue or mouse brain was prepared, using disposable equipment for each inoculum, and inoculations performed within a class 1 microbiological safety cabinet as described previously [7,10,23]. Twenty mice per group of the following four lines of mice were used. Tg(HuPrP129V^+/+^ *Prnp*^*o/o*^)-152c mice (129VV Tg152c mice) homozygous for a human PrP 129V transgene array and murine PrP null alleles (*Prnp*^*o/o*^); Tg(HuPrP129M^+/+^ *Prnp*^*o/o*^)-35c mice (129MM Tg35c mice) homozygous for a human PrP 129M transgene array and murine PrP null alleles (*Prnp*^*o/o*^); F1 cross of Tg35c and Tg152c (129MV Tg35c/Tg152c mice) to produce heterozygous 129MV mice and murine PrP null alleles (*Prnp*^*o/o*^) and wild type FVB/N mice (FVB/NHanHsd) supplied by Envigo RMS (UK) Ltd. Both Tg35c and Tg152c lines have a congenic (FVB/N *Prnp*^*o/o*^) background which is critical for prion strain differentiation and overexpress human PrP in brain at levels of 2- and 6-times that of pooled human brain, respectively. The genotype of each mouse was confirmed by PCR of ear punch DNA prior to inclusion and all mice were uniquely identified by sub-cutaneous transponders. Disposable cages were used and all cage lids and water bottles were also uniquely identified by transponder and remained with each cage of mice throughout the incubation period. Mice (female, aged 6-8 weeks) were randomly assigned to experimental groups and anaesthetised with a mixture of halothane and O_2_, and intracerebrally inoculated into the right parietal lobe with 30 µl of a 1% (w/v) tissue homogenate prepared in Dulbecco’s phosphate buffered saline (D-PBS). Thereafter all mice were examined daily for early indicators of clinical prion disease including piloerection, sustained erect ears, intermittent generalised tremor, unsustained hunched posture, rigid tail, mild loss of coordination, and clasping hind legs when lifted by the tail. Definite diagnosis of clinical prion disease (triggering experimental end point) was reached if mice exhibit any two early indicator signs in addition to one confirmatory sign, or any two confirmatory signs. The confirmatory signs included ataxia, impairment of righting reflex, dragging of hind limbs, sustained hunched posture, or significant abnormal breathing. Mice were killed (by CO_2_ asphyxiation) if exhibiting any signs of distress or once a diagnosis of prion disease was established or after post-inoculation periods of 600 days. Such elective culling after 600 days post-inoculation reduces the occurrence of ‘found dead’ mice that die of old age, in which autolytic deterioration of brain tissue often precludes IHC analyses. At post-mortem, brains from inoculated mice were removed, divided sagittally with half frozen and half fixed in formal-saline.

### Neuropathology and immunohistochemistry for detection of PrP

Transgenic or wild-type mouse brains fixed in 10% buffered formal-saline were immersed in 98% formic acid for 1 h, and following further washing in 10% buffered formal-saline, processed through graded alcohols and xylene, and paraffin wax embedded. Serial sections of 4 μm nominal thickness were taken. Deparaffinised sections were investigated for abnormal PrP deposition on a Ventana Discovery XT automated IHC staining machine (Roche Tissue Diagnostics) using protocols developed on a Ventana Benchmark staining machine [35]. Briefly, sections were treated with cell conditioning solution (Discovery CC1; Roche Tissue Diagnostics) at 95°C for either 64 min (3F4) or 96 min (6D11) followed by treatment with a low concentration of protease (Protease 3; Roche Tissue Diagnostics) for either 16 min (3F4) or 12 min (6D11). Anti-PrP monoclonal antibodies 3F4 and 6D11 were used for human or mouse PrP respectively, in conjunction with biotinylated polyclonal rabbit anti-mouse immunoglobulin secondary antibodies (Dako; Agilent) and Ventana proprietary detection reagents utilising 3,3′-diaminobenzidine tetrahydrochloride as the chromogen (DAB Map Detection Kit; Roche Tissue Diagnostics). Conventional methods on a Gemini AS Automated Slide Stainer (Thermo Fisher Scientific) were used for haematoxylin and eosin (H&E) staining. Positive controls for the staining technique were used throughout. Slides were digitally scanned on a Hamamatsu NanoZoomer s360 scanner, and images were captured from the Hamamatsu NDP.view2 software and composed with Adobe Photoshop.

## Supporting information

S1 Fig PrP^Sc^ typing in 129MV vCJD patient frontal cortex. (A) Western blot of proteinase K-digested 10% (w/v) brain homogenates (frontal cortex) from the 129MV vCJD patient and reference cases of sporadic CJD (sCJD) or 129MM vCJD using anti-PrP monoclonal antibody 3F4 and high sensitivity enhanced chemiluminescence. The provenance of each sample and the patient’s codon 129 genotype (methionine M, valine V) are shown above each lane and the propagated PrP^Sc^ type shown below (PrP^Sc^ types 2, 3 and 4; London classification, [26]). (B) Ratios of the di- and mono-glycosylated protease-resistant PrP glycoforms seen in PrP^Sc^ from the 129MV vCJD patient’s frontal cortex in comparison to PrP^Sc^ in the frontal cortex of patients with sCJD or 129MM vCJD. Where sample size is ≥ 3 symbols show mean percentage ± SEM. In some cases the error bars are smaller than the symbols used.
(TIF)

S2 Fig Patterns of spongiosis in the brains of transgenic mice challenged with 129MV vCJD patient frontal cortex.
Mice were intracerebrally inoculated with 1% (w/v) homogenate prepared from 129MV vCJD patient frontal cortex. Spongiosis in the brain of recipient infected mice was assessed on haematoxylin- and eosin-stained sections (H&E). Upper schematic drawings show the overall spatial distribution and intensity of spongiform change in the brain. Light purple shading, widely dispersed spongiosis, dark purple shading, intense focal spongiosis, yellow shading, neuronal loss. Lower panels show H&E sections demonstrating representative spongiosis in the hippocampus and cortex. Note the presence of florid plaques in the cortex of 129MM Tg35c mice. Scale bar, 100 µm for upper row (hippocampus), 50 µm for lower row (cortex).
(TIF)

S3 Fig Overview of abnormal PrP deposition and spongiosis in the brains of transgenic mice propagating type 3* PrP^Sc^. The provenance of the brain sample is designated above each column and the type of PrP^Sc^ (PrP^Sc^ type 3*; T3*) seen in infected brain is designated below. Upper schematic drawings show the overall spatial distribution and intensity of abnormal PrP deposition with red shading and PrP plaques as red dots. Lower panels show the overall spatial distribution and intensity of spongiform change. Light purple shading, widely dispersed spongiosis, dark purple shading, intense focal spongiosis, yellow shading, neuronal loss. Abnormal PrP deposition was assessed by IHC using anti PrP monoclonal antibody 3F4. Spongiosis was assessed by haematoxylin- and eosin-staining.
(TIF)

S4 Fig Western blot detection of PrP^Sc^ in 129MV vCJD patient appendix and in 129MM Tg35c transgenic mouse brains following primary transmission of 129MV vCJD patient appendix. (A-C) Western blots of proteinase K digested sodium phosphotungstic acid (NaPTA) pellets analysed with anti-PrP monoclonal antibody 3F4 using high sensitivity chemiluminescence. (A) NaPTA pellets derived from 500 µl 10% (w/v) homogenates from 129MV vCJD patient appendix or mesenteric lymph nodes, showing weak PrP^Sc^ positivity in appendix. (B) Lanes 1-5, NaPTA pellets derived from 250 µl 10% (w/v) brain homogenates from five 129MM Tg35c mice challenged with PrP^Sc^-positive 1% (w/v) 129MV vCJD patient appendix homogenate. (C) Lanes 1-5, NaPTA pellets derived from 250 µl 10% (w/v) brain homogenates from five 129MM Tg35c mice challenged with PrP^Sc^-negative 1% (w/v) 129MV vCJD patient appendix homogenate.
(TIF)

S1 Table Summary of historical transmissions of prions from 129MM vCJD brain to transgenic and wild-type mice. (PDF)

S2 Table Primary transmission of prions from 129MV vCJD patient brain regions to transgenic and wild-type mice. (PDF)

S3 Table Secondary transmission of prions from 129MV vCJD patient frontal cortex to transgenic and wild-type mice. (PDF)

S4 Table Primary transmission of prions from 129MV vCJD patient appendix and mesenteric lymph node to transgenic and wild-type mice. (PDF)

S5 Table Primary transmission of prions from 129MV vCJD spleen pools to transgenic mice. (PDF)

S1 Data Images of original western blot autoradiography films. (PDF)

S2 Data PrP glycoform ratios of PrP^Sc^ types. (XLSX)
