# Supplementary figures and images for: Isolation of a novel human prion strain from a PRNP codon 129 heterozygous vCJD patient
Source: PLoS Pathog. 2025 Feb 20;21(2):e1012904. doi: 10.1371/journal.ppat.1012904 (PMC11841882; doi:10.1371/journal.ppat.1012904)

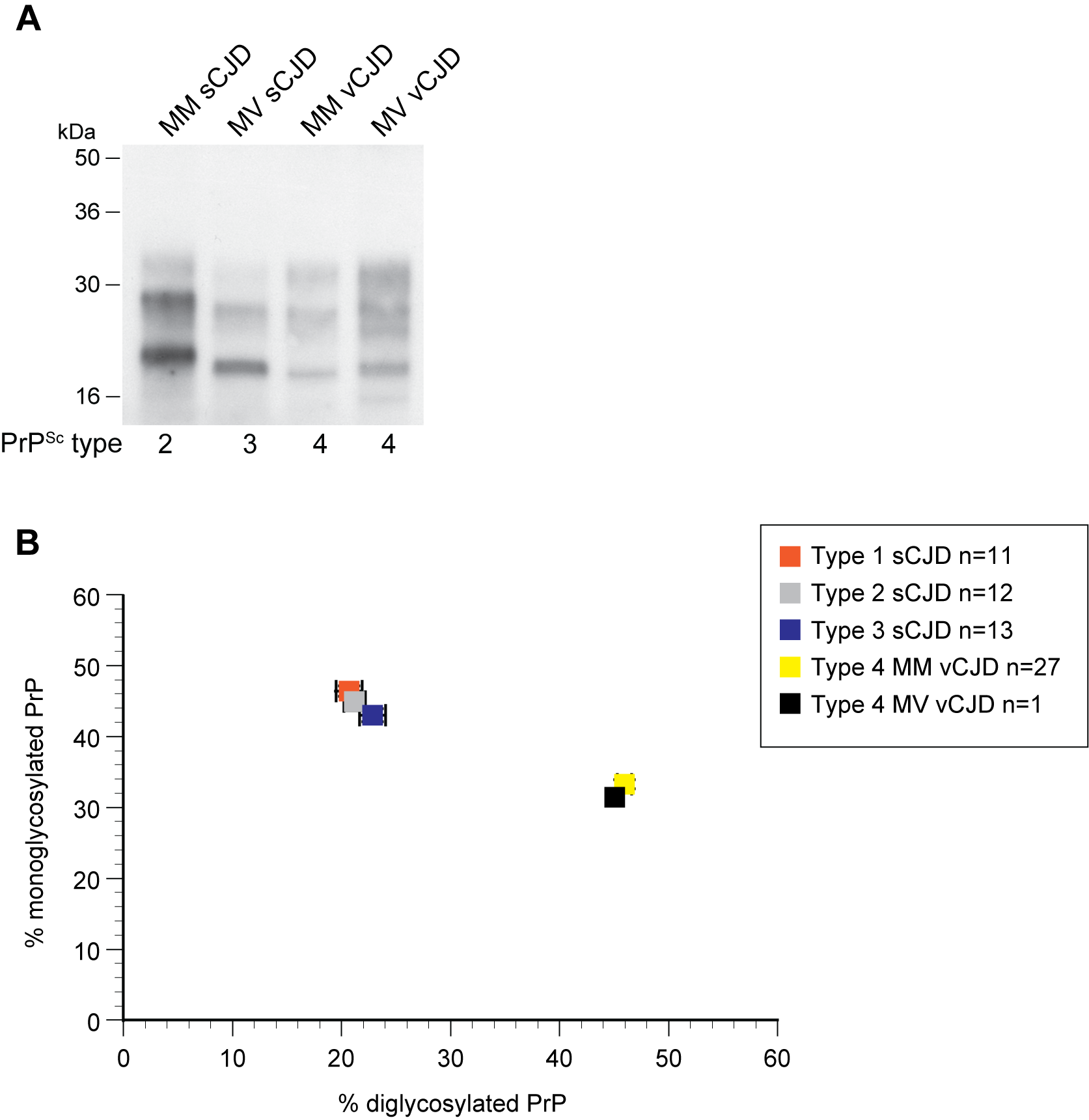

Supplement: S1 Fig — (A) Western blot of proteinase K-digested 10% (w/v) brain homogenates (frontal cortex) from the 129MV vCJD patient and reference cases of sporadic CJD (sCJD) or 129MM vCJD using anti-PrP monoclonal antibody 3F4 and high sensitivity enhanced chemiluminescence. The provenance of each sample and the patient’s codon 129 genotype (methionine M, valine V) are shown above each lane and the propagated PrPSc type shown below (PrPSc types 2, 3 and 4; London classification, [26]). (B) Ratios of the di- and mono-glycosylated protease-resistant PrP glycoforms seen in PrPSc from the 129MV vCJD patient’s frontal cortex in comparison to PrPSc in the frontal cortex of patients with sCJD or 129MM vCJD. Where sample size is ≥ 3 symbols show mean percentage ± SEM. In some cases the error bars are smaller than the symbols used. (TIF) [file ppat.1012904.s001.tif]

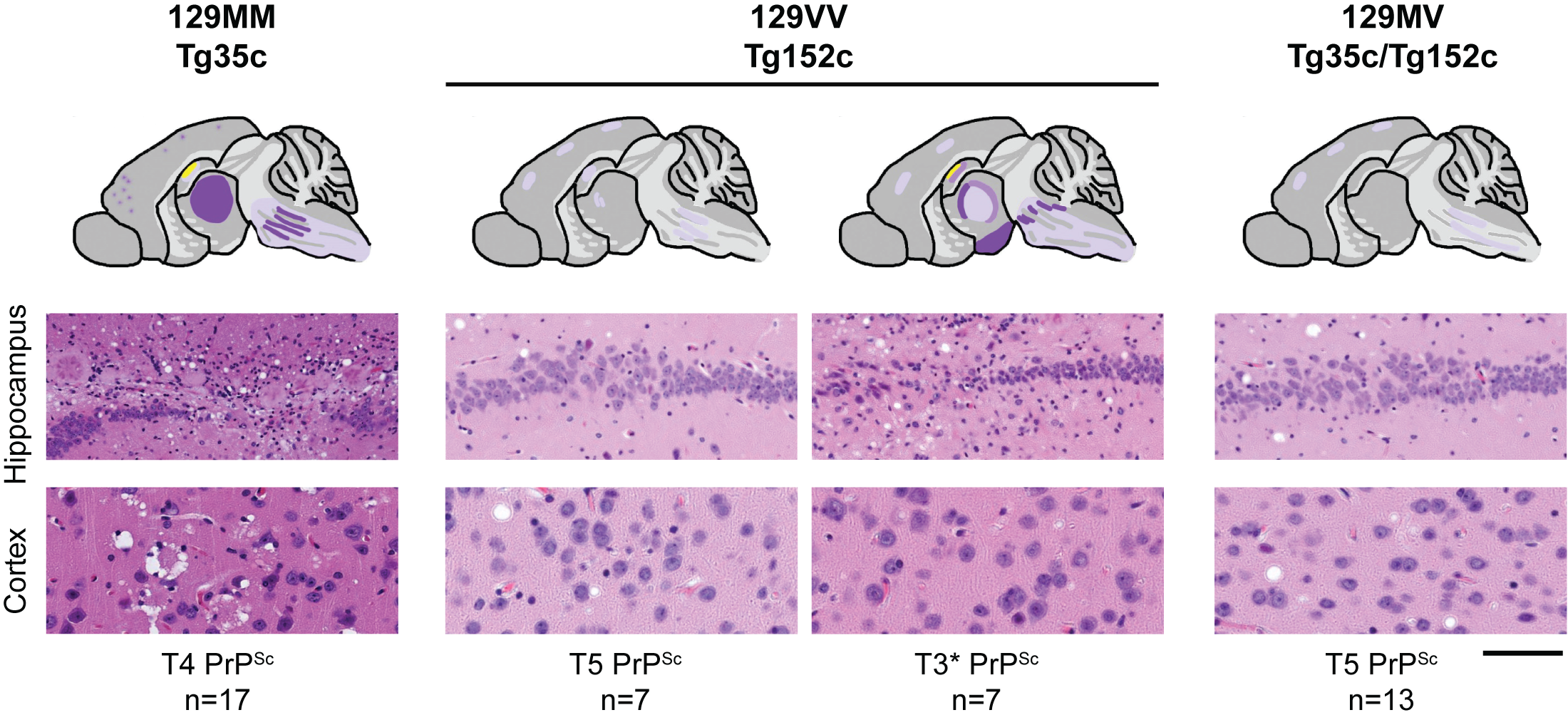

Supplement: S2 Fig — Mice were intracerebrally inoculated with 1% (w/v) homogenate prepared from 129MV vCJD patient frontal cortex. Spongiosis in the brain of recipient infected mice was assessed on haematoxylin- and eosin-stained sections (H&E). Upper schematic drawings show the overall spatial distribution and intensity of spongiform change in the brain. Light purple shading, widely dispersed spongiosis, dark purple shading, intense focal spongiosis, yellow shading, neuronal loss. Lower panels show H&E sections demonstrating representative spongiosis in the hippocampus and cortex. Note the presence of florid plaques in the cortex of 129MM Tg35c mice. Scale bar, 100 µm for upper row (hippocampus), 50 µm for lower row (cortex). (TIF) [file ppat.1012904.s002.tif]

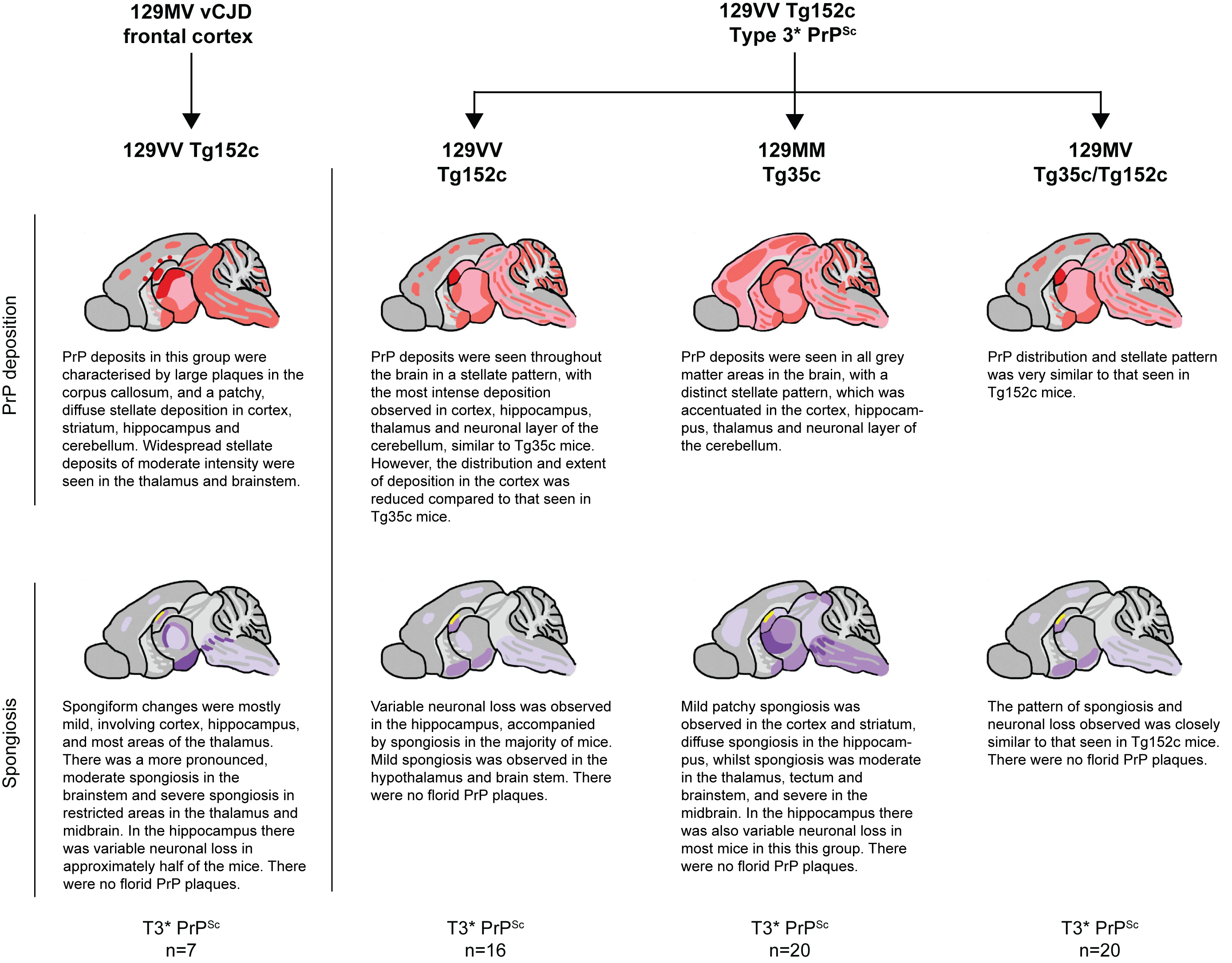

Supplement: S3 Fig — The provenance of the brain sample is designated above each column and the type of PrPSc (PrPSc type 3*; T3*) seen in infected brain is designated below. Upper schematic drawings show the overall spatial distribution and intensity of abnormal PrP deposition with red shading and PrP plaques as red dots. Lower panels show the overall spatial distribution and intensity of spongiform change. Light purple shading, widely dispersed spongiosis, dark purple shading, intense focal spongiosis, yellow shading, neuronal loss. Abnormal PrP deposition was assessed by IHC using anti PrP monoclonal antibody 3F4. Spongiosis was assessed by haematoxylin- and eosin-staining. (TIF) [file ppat.1012904.s003.tif]

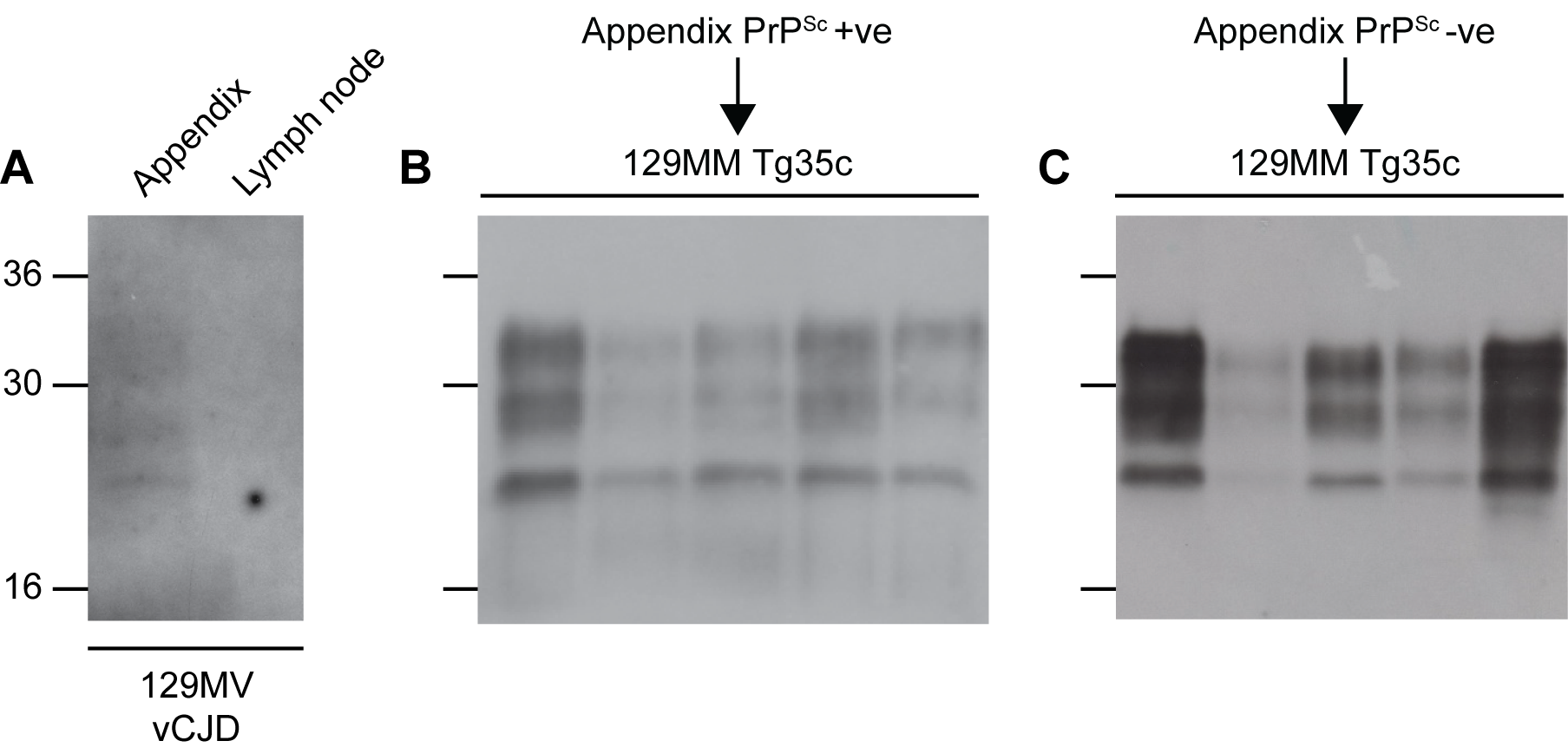

Supplement: S4 Fig — (A-C) Western blots of proteinase K digested sodium phosphotungstic acid (NaPTA) pellets analysed with anti-PrP monoclonal antibody 3F4 using high sensitivity chemiluminescence. (A) NaPTA pellets derived from 500 µl 10% (w/v) homogenates from 129MV vCJD patient appendix or mesenteric lymph nodes, showing weak PrPSc positivity in appendix. (B) Lanes 1-5, NaPTA pellets derived from 250 µl 10% (w/v) brain homogenates from five 129MM Tg35c mice challenged with PrPSc-positive 1% (w/v) 129MV vCJD patient appendix homogenate. (C) Lanes 1-5, NaPTA pellets derived from 250 µl 10% (w/v) brain homogenates from five 129MM Tg35c mice challenged with PrPSc-negative 1% (w/v) 129MV vCJD patient appendix homogenate. (TIF) [file ppat.1012904.s004.tif]
