# Supplementary material for: Isolation of a novel human prion strain from a PRNP codon 129 heterozygous vCJD patient
Source: PLoS Pathog. 2025 Feb 20;21(2):e1012904. doi: 10.1371/journal.ppat.1012904 (PMC11841882; doi:10.1371/journal.ppat.1012904)
Supplement: S1 Table — (PDF) [file ppat.1012904.s005.pdf]

S1 Table. Summary of historical transmissions of prions from 129MM vCJD brain to transgenic and wild-type mice.

Inoculum	Mouse line			
	**Human PrP 129MM Tg35, Tg35c, Tg45** [a]	**Human PrP 129VV Tg152, Tg152c** [b]	**Human PrP 129MV Tg45/Tg152** [c]	**Wild-type FVB/N** [d]
129MM vCJD patient brain with type 4 PrP^{Sc}	High rates of infection, typically 100%, low incidence of clinical prion disease<br><br>Propagation of type 4 PrP^{Sc}<br><br>Recapitulation of vCJD neuropathology with abundant florid PrP plaques in cortex<br><br>References [7–9,23–25]	Presence of a transmission barrier, typical infection rates <100%, low incidence of clinical prion disease<br><br>Propagation of type 5 PrP^{Sc}<br><br>Deposition of irregular, non-florid PrP plaques in corpus callosum, minimal PrP deposition elsewhere in brain<br><br>References [6,8,10,11,25]	High rates of infection, typically 100%, no clinical prion disease<br><br>Propagation of type 4 PrP^{Sc}<br><br>Deposition of non-florid PrP plaques in the corpus callosum, diffuse synaptic PrP deposition and occasional small non-florid PrP plaques in brainstem and thalamus<br><br>References [9]	Presence of a transmission barrier, typical infection rates <100%, infected mice usually have clinical prion disease<br><br>Propagation of di-glycosylated PrP dominant PrP^{Sc}<br><br>Patchy diffuse and granular PrP deposits.<br>No florid plaques<br><br>References [6,8,10,11,25]

[a] Brain levels of expression of human PrP in 129MM Tg35, 129MM Tg35c and 129MM Tg45 mice are 2-fold, 2-fold or 4-fold higher than a pooled normal human brain standard, respectively.
[b] Brain levels of expression of human PrP in 129VV Tg152 and 129VV Tg152c mice are 6-fold higher than a pooled normal human brain standard.
[c] Ratio of human PrP 129M:129V in Tg45/152 mice is 2:3.
[d] FVB/N mice are homozygous for *Prnp* allele a.
