# Supplementary material for: Isolation of a novel human prion strain from a PRNP codon 129 heterozygous vCJD patient
Source: PLoS Pathog. 2025 Feb 20;21(2):e1012904. doi: 10.1371/journal.ppat.1012904 (PMC11841882; doi:10.1371/journal.ppat.1012904)
Supplement: S2 Table — (PDF) [file ppat.1012904.s006.pdf]

S2 Table. Primary transmission of prions from 129MV vCJD patient brain regions to transgenic and wild-type mice

Brain Region [a]	Mouse line	Total attack rate [b]	Clinical attack rate [c]	Mean incubation period (days ± SEM) [d]	Survival periods of subclinically infected mice (days) [e]
Frontal Cortex	129MM Tg35c	17/17	4/17	387 ± 65	362, 385, 420, 452, 537, 537, 627 (7)
	129VV Tg152c	14/15	8/15	444 ± 18	439, 452, 558, 627 (3)
	129MV Tg35c/152c	13/15	2/15	502, 525	374, 493, 525, 535, 578, 578, 627 (5)
	FVB/N	18/18	18/18	410 ± 9	NA
Parietal Cortex	129MM Tg35c	19/19	2/19	399, 433	497, 509, 532, 560, 580, 615 (12)
	129VV Tg152c	17/19	8/19	508 ±19	426, 615 (8)
	129MV Tg35c/152c	18/18	6/18	529 ± 29.33	615 (12)
	FVB/N	19/19	19/19	414 ± 13	NA
Cerebellum	129MM Tg35c	20/20	1/20	256	212, 337, 557, 603 (16)
	129VV Tg152c	19/19	2/19	487, 574	309, 471, 476, 490, 504, 604 (12)
	129MV Tg35c/152c	20/20	2/20	521, 588	351, 483, 589, 604 (15)
	FVB	20/20	19/20	411 ± 10	305

Thalamus	129MM Tg35c	19/19	1/19	471	509, 524, 562, 602 (15)
	129VV Tg152c	17/19	3/19	542 ± 7	504, 602 (13)
	129MV Tg35c/152c	19/19	2/19	455, 497	376, 602 (16)
	FVB	19/19	18/19	459 ± 16	602 (1)

[a] All mice were inoculated with 30 µl of 1% (w/v) 129MV vCJD patient tissue homogenate.
[b] Total attack rate is defined as the total number of clinically affected and subclinically infected mice as a proportion of the number of inoculated mice. Subclinical prion infection was assessed by immunohistochemical examination of brain for abnormal PrP deposition and immunoblot analysis of brain homogenate for detectable PrP^{Sc} (by direct western blotting or after NaPTA precipitation if required).
[c] Clinical attack rate is defined as the total number of clinically affected mice as a proportion of the number of inoculated mice.
[d] Incubation periods are reported for clinically affected mice in days; where n ≥ 3 the mean ± SEM is reported otherwise individual incubation times are given.
[e] Survival periods of subclinically infected mice reports the number of days between inoculation and culling due to inter-current illness or termination of the experiment. The survival periods of individual mice culled before termination of the experiment are shown together with the survival period at termination of the experiment, with number of mice culled at this point shown in parentheses.
NA, not applicable.
