# Supplementary material for: Isolation of a novel human prion strain from a PRNP codon 129 heterozygous vCJD patient
Source: PLoS Pathog. 2025 Feb 20;21(2):e1012904. doi: 10.1371/journal.ppat.1012904 (PMC11841882; doi:10.1371/journal.ppat.1012904)
Supplement: S3 Table — (PDF) [file ppat.1012904.s007.pdf]

S3 Table. Secondary transmission of prions from 129MV vCJD patient frontal cortex to transgenic and wild-type mice

Inoculum [a]	Mouse line	Total attack rate [b]	Clinical attack rate [c]	Mean incubation period (days ± SEM) [d]	Survival periods of subclinically infected mice (days) [e]
129MM Tg35c propagating PrP^{Sc} type 4	129MM Tg35c	18/18	3/18	523 ± 18	524, 607 (14)
	129VV Tg152c	11/18	0/18	NA	519, 520, 610 (9)
	129MV Tg35c/152c	5/19	0/19	NA	541, 604, 610 (3)
129VV Tg152c propagating PrP^{Sc} type 3* with novel stellate PrP neuropathology	129MM Tg35c	20/20	20/20	276 ± 3	NA
	129VV Tg152c	16/16	16/16	289 ± 6	NA
	129MV Tg35c/152c	20/20	20/20	321 ± 6	NA
	FVB/N	17/18	15/18	387 ± 17	342, 348
129VV Tg152c propagating PrP^{Sc} type 5 with PrP plaques	129MM Tg35c	19/20	5/20	505 ± 14	533, 608, 615 (12)
	129VV Tg152c	15/20	3/20	526 ± 39	513, 622 (11)
	129MV Tg35c/152c	12/18	0/18	NA	622 (12)
	FVB	16/20	11/20	525 ± 13	411, 468, 624 (3)
129MV Tg35c/152c	129MM Tg35c	17/17	2/17	384, 516	509, 512, 609 (13)

propagating PrP^{Sc} type 5 with PrP plaques	129VV Tg152c	11/14	3/14	569 ± 21	610 (8)
	129MV Tg35c/152c	14/19	3/19	509 ± 54	595, 614 (10)
	FVB	11/14	9/14	501 ± 23	392, 614 (1)

[a] All mice were inoculated with 30 µl of 1% (w/v) transgenic mouse brain homogenate.
[b] Total attack rate is defined as the total number of clinically affected and subclinically infected mice as a proportion of the number of inoculated mice. Subclinical prion infection was assessed by immunohistochemical examination of brain for abnormal PrP deposition and immunoblot analysis of brain homogenate for detectable PrP^{Sc} (by direct western blotting or after NaPTA precipitation if required).
[c] Clinical attack rate is defined as the total number of clinically affected mice as a proportion of the number of inoculated mice.
[d] Incubation periods are reported for clinically affected mice in days; where $n \geq 3$ the mean ± SEM is reported otherwise individual incubation times are given.
[e] Survival periods of subclinically infected mice reports the number of days between inoculation and culling due to inter-current illness or termination of the experiment. The survival periods of individual mice culled before termination of the experiment are shown together with the survival period at termination of the experiment, with number of mice culled at this point shown in parentheses.
NA, not applicable.
