# Supplementary material for: Isolation of a novel human prion strain from a PRNP codon 129 heterozygous vCJD patient
Source: PLoS Pathog. 2025 Feb 20;21(2):e1012904. doi: 10.1371/journal.ppat.1012904 (PMC11841882; doi:10.1371/journal.ppat.1012904)
Supplement: S4 Table — (PDF) [file ppat.1012904.s008.pdf]

**S4 Table. Primary transmission of prions from 129MV vCJD patient appendix and mesenteric lymph node to transgenic and wild-type mice**

| Tissue [a] | Mouse line | Total attack rate [b] | Clinical attack rate [c] | Incubation period (days) [d] | Survival periods of subclinically infected mice (days) [e] |
|---|---|---|---|---|---|
| Appendix $PrP^{Sc}$ +ve | 129MM Tg35c | 16/19 | 0/19 | NA | 489, 607 (15) |
| | 129VV Tg152c | 9/19 | 0/19 | NA | 489, 608 (8) |
| | 129MV Tg35c/152c | 9/20 | 0/20 | NA | 609 (9) |
| | FVB/N | 0/19 | 0/19 | NA | NA |
| Appendix $PrP^{Sc}$ -ve | 129MM Tg35c | 20/20 | 2/20 | 527, 533 | 576, 581, 608 (16) |
| | 129VV Tg152c | 5/19 | 0/19 | NA | 609 (5) |
| | 129MV Tg35c/152c | 9/19 | 0/19 | NA | 611 (9) |
| | FVB/N | 2/18 | 2/18 | 577, 596 | NA |
| Mesenteric lymph nodes (pool) $PrP^{Sc}$ -ve | 129MM Tg35c | 20/20 | 0/20 | NA | 492, 492, 548, 583, 608 (16) |
| | 129VV Tg152c | 7/19 | 1/19 | 489 | 497, 540, 609 (4) |
| | 129MV Tg35c/152c | 13/18 | 2/18 | 488, 575 | 528, 587, 610 (9) |
| | FVB/N | 1/20 | 0/20 | NA | 611 (1) |

[a] All mice were inoculated with 30 µl of 1% (w/v) 129MV vCJD patient tissue homogenate.
[b] Total attack rate is defined as the total number of clinically affected and subclinically infected mice as a proportion of the number of inoculated mice. Subclinical prion infection was assessed by immunohistochemical examination of brain for abnormal PrP deposition and immunoblot analysis for detectable $PrP^{Sc}$ after NaPTA precipitation of 250 µl 10% (w/v) brain homogenate.
[c] Clinical attack rate is defined as the total number of clinically affected mice as a proportion of the number of inoculated mice.
[d] Individual incubation periods are reported for clinically affected mice in days.
[e] Survival periods of subclinically infected mice reports the number of days between inoculation and culling due to inter-current illness or termination of the experiment. The survival periods of individual mice culled before termination of the experiment are shown together with the survival period at termination of the experiment, with number of mice culled at this point shown in parentheses.
NA, not applicable.
