# Supplementary material for: Isolation of a novel human prion strain from a PRNP codon 129 heterozygous vCJD patient
Source: PLoS Pathog. 2025 Feb 20;21(2):e1012904. doi: 10.1371/journal.ppat.1012904 (PMC11841882; doi:10.1371/journal.ppat.1012904)
Supplement: S5 Table — (PDF) [file ppat.1012904.s009.pdf]

S5 Table. Primary transmission of prions from 129MV vCJD spleen pools to transgenic mice

Tissue [a]	Mouse line	Total attack rate [b]	Incubation period (days)	Survival periods of subclinically infected mice (days) [c]
Spleen (pool A) PrP^{Sc} -ve	129MM Tg35c	0/20	NA	NA
Spleen (pool B) PrP^{Sc} -ve	129MM Tg35c	1/20	NA	602 [d]
Spleen (pool C) PrP^{Sc} -ve	129MM Tg35c	0/17	NA	NA

[a] All mice were inoculated with 30 µl of 1% (w/v) 129MV vCJD tissue homogenate.
[b] Total attack rate is defined as the total number of clinically affected and subclinically infected mice as a proportion of the number of inoculated mice. Subclinical prion infection was assessed by immunohistochemical examination of brain for abnormal PrP deposition and immunoblot analysis for detectable PrP^{Sc} after NaPTA precipitation of 250 µl 10% (w/v) brain homogenate.
[c] Survival periods of subclinically infected mice reports the number of days between inoculation and culling due to inter-current illness or termination of the experiment.
[d] The single subclinically infected mouse was culled at termination of the experiment. The brain showed weak propagation of diglycosylated PrP dominant PrP^{Sc} (that required NaPTA precipitation for detection) and the presence of two PrP microplaques in the cortex and midbrain by IHC.
NA, not applicable.
