# Supplementary material for: Isolation of a novel human prion strain from a PRNP codon 129 heterozygous vCJD patient
Source: PLoS Pathog. 2025 Feb 20;21(2):e1012904. doi: 10.1371/journal.ppat.1012904 (PMC11841882; doi:10.1371/journal.ppat.1012904)
Supplement: S1 Data — (PDF) [file ppat.1012904.s010.pdf]

S1 Data. Images of original western blot autoradiography films

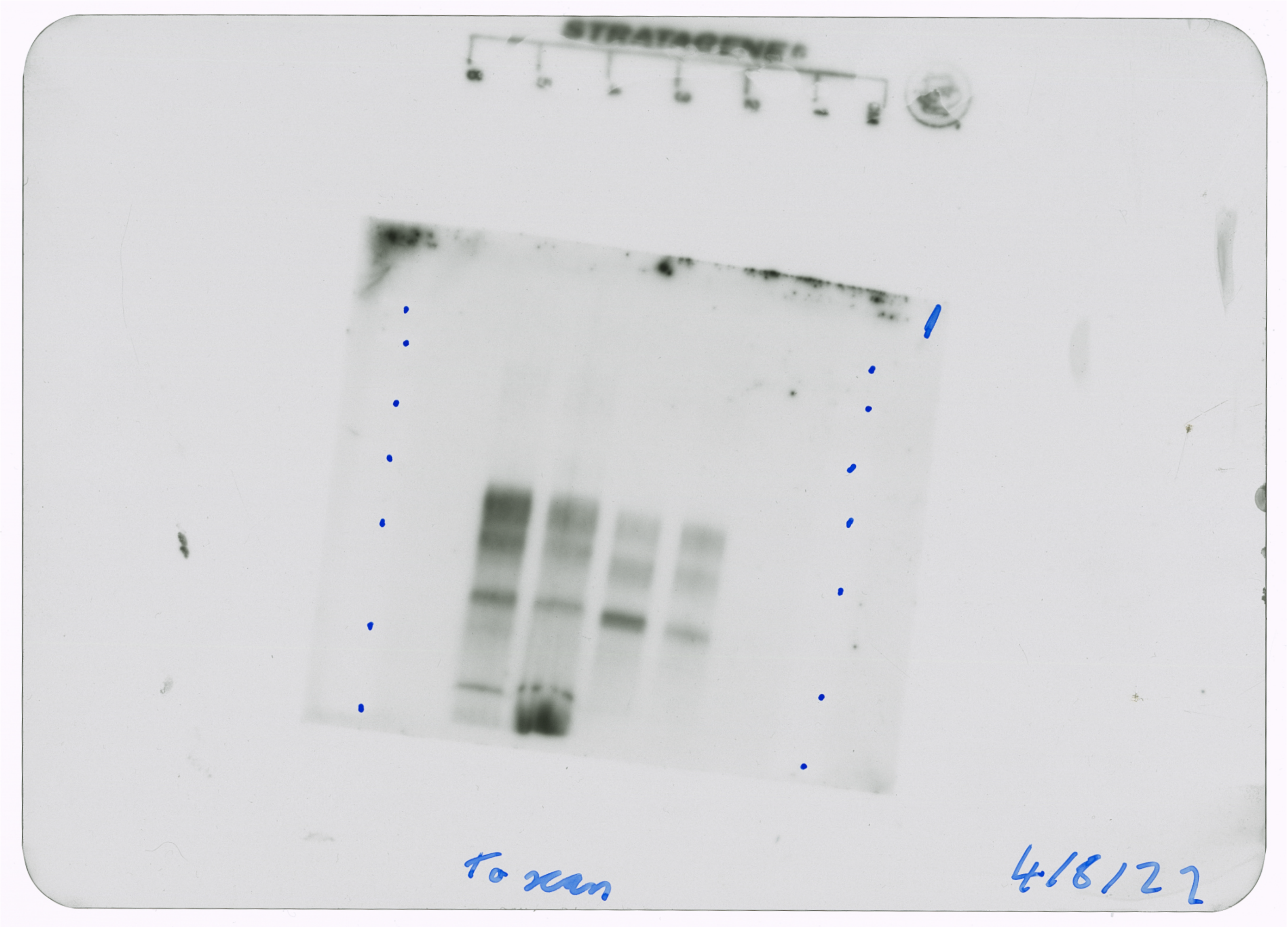

Figure 2

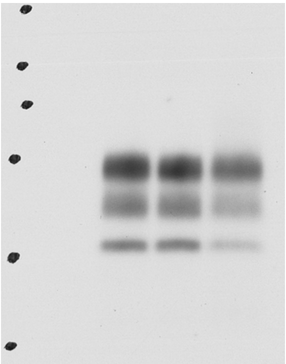

Figure 3

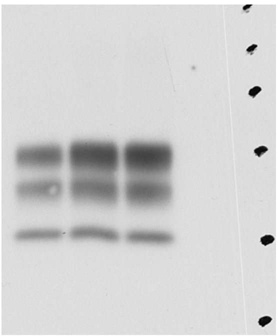

Figure 6

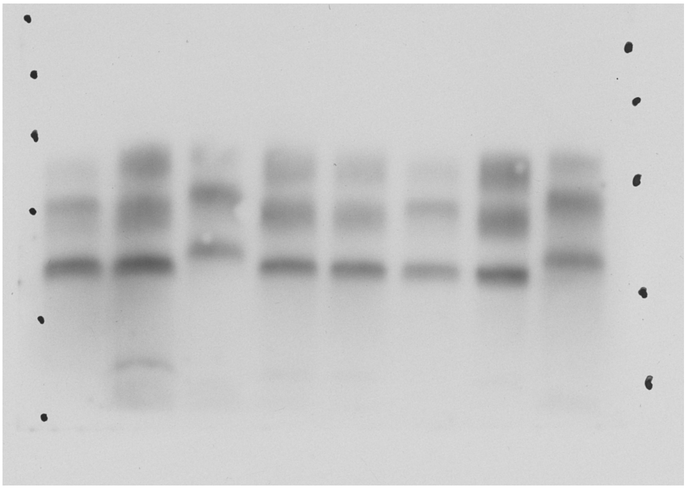

Figure 8

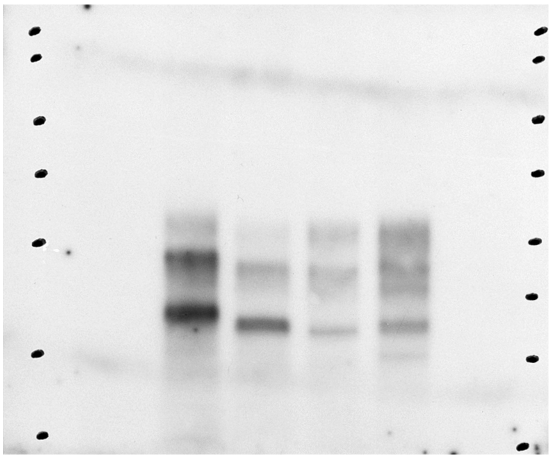

S1 figure

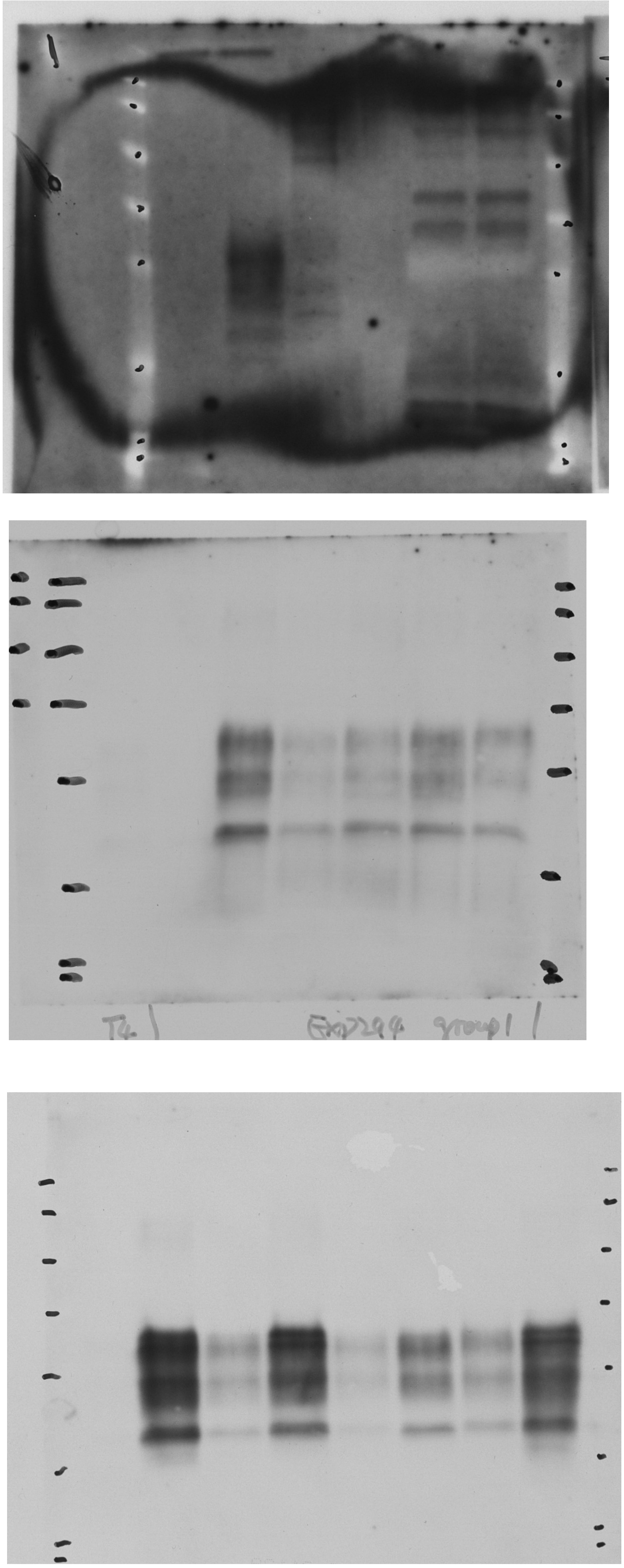

A

B

C

S4 figure
